# Outcomes of Transcatheter Aortic Valve Implantation with Abbott’s Portico Compared to Edwards’ SAPIEN 3: A Systematic Review and Meta-Analysis

**DOI:** 10.3390/jcm15103573

**Published:** 2026-05-07

**Authors:** Mirosław Gozdek, Mariusz Kowalewski, Tomasz Urbanowicz, Giuseppe Maria Raffa

**Affiliations:** 1 Thoracic Research Centre, Innovative Medical Forum, Collegium Medicum in Bydgoszcz, Nicolaus Copernicus University, 85067 Bydgoszcz, Poland; 2 Department of Cardiac Surgery and Transplantology, National Medical Institute of the Ministry of Interior, 02507 Warsaw, Poland; 3 Cardio-Thoracic Surgery Department, Heart and Vascular Centre, Maastricht University Medical Centre, 6229 HX Maastricht, The Netherlands; 4 Cardiac Surgery and Transplantology Department, Poznań University of Medical Sciences, 61701 Poznań, Poland; 5 IRCCS-ISMETT, 90127 Palermo, Italy; 6 Cardiac Surgery, Department of Precision Medicine in Medical Surgical and Critical Area (Me.Pre.C.C.), University of Palermo, 90134 Palermo, Italy

**Keywords:** meta-analysis, TAVI, Portico, SAPIEN 3, heart failure

## Abstract

**Background/Objectives**: Nowadays, transcatheter aortic valve implantation (TAVI) is widespread in patients with severe aortic valve stenosis. New prosthesis designs are becoming available to address the shortcomings of their predecessors and improve clinical outcomes. **Methods**: Electronic databases were screened for studies comparing outcomes of TAVI with Portico and SAPIEN 3. In a random-effects meta-analysis the pooled incidence rates of procedural, clinical and functional outcomes, according to VARC-2 definitions, were assessed. **Results**: Thirteen observational studies and one multi-center randomized clinical trial enrolling 20,522 patients (Portico N = 3001 and SAPIEN 3 N = 17,521) were included in the analysis. The need for more than one prosthesis during initial implantation was significantly higher among Portico recipients compared to SAPIEN 3 recipients: (RR 2.72 [1.36, 5.45] *p* = 0.005). Pre- and post-dilatation were performed more frequently in the Portico group (RR 1.53 [1.12, 2.09], *p* = 0.008 and RR 4.21 [2.83, 6.26], *p* < 0.00001, respectively). Moderate-to-severe paravalvular leak (PVL) was significantly more common in the Portico arm (RR 3.27 [1.80, 5.91] *p* < 0.0001). In contrast, the mean gradient and rate of prosthesis–patient mismatch (PPM) was significantly lower in the Portico group (MD −31.58 [−37.02; −26.14] mmHg and RR 0.42 [0.32, 0.55], *p* < 0.00001). Recipients of Portico demonstrated over 60% higher risk of permanent pacemaker implantation (PPI) compared to SAPIEN 3 (RR 1.62 [1.25, 2.10], *p* = 0.0002). Other procedural and short-term clinical outcomes, including neurologic events, major vascular complications, life threatening or major bleeding, acute kidney injury, myocardial infarction and mortality did not differ between the devices. A difference in mortality was observed at the 1-year follow-up (RR 1.26 [1.06, 1.51], *p* = 0.01; *I*^2^ = 5%). **Conclusions**: The evidence shows good short-term outcomes for both valves. Compared to SAPIEN 3, Portico was associated with a significantly higher rate of moderate-to-severe PVL and PPI, but a lower mean gradient and incidence of PPM. A significantly higher 1-year mortality was observed in the Portico group.

## 1. Introduction

We are witnessing a constant increase in the number of patients with aortic valve stenosis treated with transcatheter aortic valve implantation (TAVI). The technique is now considered an alternative to surgical treatment and is recommended not only for inoperable high-risk patients but also for those at intermediate and even low risk [1,2]. Furthermore, there is a consistently growing number of TAVI-treated patients with other aortic valve defects leading to chronic and acute heart failure, including severe non-tricuspid valve stenosis and isolated severe regurgitation, conditions that were previously reserved for surgery. We have also observed increasing patient awareness regarding the availability of less invasive treatments for aortic valve disease, which has contributed to a rise in TAVI requests. Over the past two decades, the primary question has been “when TAVI?” Today, the question appears to be “how TAVI?”.

In the early years after the introduction of TAVI in 2002, two types of transcatheter devices were available to physicians: the balloon-expandable SAPIEN (Edwards Lifesciences, Irvine, CA, USA) and the self-expanding CoreValve (Medtronic, Minneapolis, MN, USA). Although these early-generation valves provided good clinical outcomes, they were not without disadvantages. These included vascular complications, bleeding, stroke, conduction disturbances requiring permanent pacemaker implantation (PPI), prosthesis–patient mismatch (PPM), and paravalvular leak (PVL). The latter is a major concern, as it is associated with adverse clinical events and increased late mortality compared to surgery [3,4]. Technological advancements have led to improvements in existing devices and to the development of new ones, aiming to minimize the drawbacks of first-generation transcatheter valves.

Currently, in the emerging era of personalized and precision medicine for patients with advanced chronic heart valve disease, studies comparing different TAVI prostheses across various patient populations and anatomical settings are needed to ensure maximum procedural safety, achieve optimal clinical and functional outcomes, and provide guidance for the development of new devices.

The aim of this investigation was to evaluate and compare the outcomes of TAVI using the self-expanding, intra-annular Portico (Abbott, Santa Clara, CA, USA) and the balloon-expandable, intra-annular SAPIEN 3, both next-generation devices, in patients with symptomatic severe aortic stenosis. A summary of the valve characteristics is available in Table 1.

## 2. Materials and Methods

### 2.1. Data Sources and Search Strategy

This study was performed in accordance with the MOOSE statement and PRISMA guidelines [5,6]. The MOOSE/PRISMA checklist is available in Supplementary Tables S1 and S2. We searched PubMed, Google Scholar, Web of Science and Scopus until October 2025. The search terms were: Portico and/or SAPIEN 3 and transcatheter valve or transcatheter aortic valve. Only peer-reviewed articles published in English were considered. The reference lists of included original studies were manually reviewed and cross-verified.

### 2.2. Selection Criteria and Quality Assessment

Studies were included if they met all of the following criteria:

- Human study
- Study or study arms comparing the strategy of transcatheter aortic valve implantation with Portico and SAPIEN 3 directly or indirectly

Studies were excluded if they were:

- An in-vitro study
- A single arm study
- Outcomes of interest were not reported

No restrictions regarding the number of patients included or characteristics of the population were imposed.

Two reviewers (M.G. and M.K.) carried out the study selection process, extracting relevant patient and study characteristics, as well as outcomes of interest. The same two authors independently evaluated trial eligibility and risk of bias. Disagreements were settled through consensus. The methodological quality of randomized controlled trials (RCTs) was evaluated using the RoB 2 tool (Cochrane risk of bias tool version 2) [7], whereas non-randomized studies were appraised using ROBINS-I (Risk of Bias in Non-Randomized Studies of Interventions), which is designed to assess bias in cohort studies included in systematic reviews and meta-analyses [8].

### 2.3. Endpoint Selection

Outcome measures were defined according to the Valve Academic Research Consortium-2 (VARC-2) criteria [9]. Procedural endpoints of interest included: a requirement for more than one prosthesis and a composite of other TAVI-related complications (such as conversion to open surgery, coronary obstruction, ventricular septal perforation, damage or dysfunction of the mitral valve apparatus, endocarditis, cardiac tamponade, prosthetic valve thrombosis or malpositioning, migration, embolization, or ectopic deployment). Pre- and post-dilation—non-VARC endpoints—were also examined as factors that could influence clinical outcomes such as stroke and pacemaker implantation.

Early (up to 30 days) clinical endpoints comprised: life-threatening and major bleeding, major vascular complications (MVCs), neurological events (stroke and/or transient ischemic attack), myocardial infarction (MI), permanent pacemaker implantation (PPI), acute kidney injury (AKI), and mortality. The functional outcomes assessed included: moderate-to-severe paravalvular leak (PVL), mild PVL, mean trans-prosthetic gradient, prosthetic effective orifice area (EOA), and prosthesis–patient mismatch (PPM). Outcomes recorded during the one-year follow-up period were: rehospitalization due to heart failure, neurological events, MI, and death.

### 2.4. Statistical Analysis

For dichotomous outcomes, risk ratios (RRs) with corresponding 95% confidence intervals (95% CI) served as the main summary statistics. For continuous outcomes, mean differences (MDs) and 95% CIs were calculated using a random-effects model. To address the limited power of the Cochran Q test, heterogeneity was quantified using the *I*^2^ statistic, defined as *I*^2^ = [(Q − df)/Q] × 100%, where Q is the chi-squared value and df it the degrees of freedom [10]. This metric indicates the percentage of variation across studies not attributable to chance. *I*^2^ values below 40% suggest no substantial heterogeneity; values between 40% and 70% point to moderate heterogeneity; and values exceeding 70% reflect high heterogeneity. Given the anticipated high degree of heterogeneity among mostly non-randomized trials, an inverse-variance random-effects model (DerSimonian-Laird) was applied as a more conservative approach that accounts for both within-study and between-study variability. Baseline characteristics were compared using weighted means for continuous variables and pooled proportions for categorical variables, with between-group differences assessed using Student’s *t*-test and chi-square test, respectively.

When a study reported median values with interquartile ranges rather than means and standard deviations (SDs), the latter were estimated using the method described by Wan and colleagues [11]. In cases where both arms reported zero events, a sensitivity analysis was conducted using risk difference (RD) with a corresponding 95% CI. All statistical computations were performed using Review Manager 5.4.1 (The Cochrane Collaboration, 2020, London, UK). A two-sided *p*-value ≤ 0.05 was considered statistically significant, with no adjustment made for multiple comparisons.

## 3. Results

### 3.1. Study Selection and Bias

**Figure 1:**
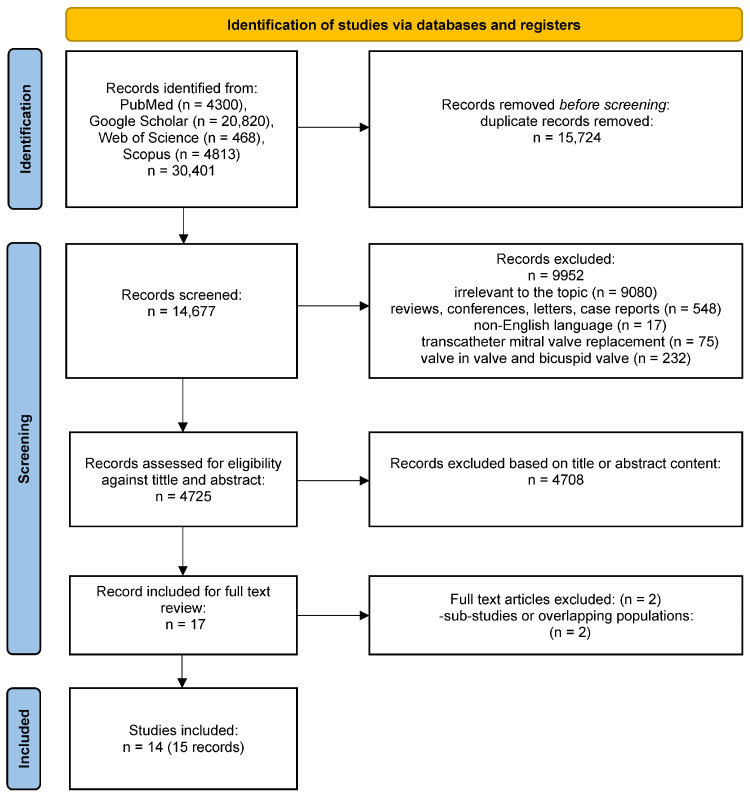
PRIMSA flowchart of the study selection process for the meta-analysis.

The study selection process and reasons for the exclusion of some studies are described in Figure 1. A systematic search of the online databases allowed the collection of 30,401 potentially eligible records that were retrieved for scrutiny. Of those, 30,385 were further excluded because they were not pertinent to the design of the meta-analysis or did not meet the explicit inclusion criteria. Thirteen observational studies (among them eight multicenter registries, two propensity score-matched and two propensity score-weighted) along with one multi-center randomized clinical trial [12,13,14,15,16,17,18,19,20,21,22,23,24,25,26] enrolling N = 20,522 patients were eventually included in the analysis. Potential sources of the studies’ bias were analyzed with the use of components recommended by the ROBINS-I, as well as RoB2 tools, and the results are shown in Supplementary Table S3. Overall, the retrospective studies reported moderate risk of bias. Most commonly biases arose from participant selection for the study by designated heart teams and subjective distribution of the participants within the study arms by designated operators. The RCT registered a low risk of bias. The study characteristics, as well as baseline patient and procedural characteristics, are reported in Table 2 and Table 3.

Supplementary Table S4 lists the selection criteria for the procedure and valve, as well as inclusion and exclusion criteria within particular studies. Details on the baseline characteristics of the patients and the procedure are available in Supplementary Tables S5 and S6.

In the study conducted by Abdelshafy et al. [12], data on PVL comes from the analysis of aortograms. The study by Brown et al. [13] compared Portico to SAPIEN 3 Ultra prosthesis built on the SAPIEN 3 platform, and the only difference was a 40% taller external seal of Ultra. Giordano et al. [16] and Corcione et al. [14] reported data from the RISPEVA study at 30-day and 1-year follow-up, respectively. In the RISPEVA study TAVI was also performed for degenerated surgical bioprostheses (11.5% Portico, 2.6% SAPIEN 3), mixed valvular disease (4.3% Portico, 9.8% SAPIEN 3), and isolated aortic regurgitation (0.3% Portico, 0.4% SAPIEN 3). The study by Kim et al. [17], as well as Okuno et al. [21], focused on comparing prostheses according to the extent of native valve calcification. The studies by Leone et al. [18] and Voigtländer et al. [26] compared different prosthetic valves implanted in patients with a small aortic annulus. Slight overlap is possible between the studies conducted by Costa [15] and Leone [18], Giordano [15] and Leone [18], as well as between Rudolph [23] and other studies conducted in Germany (Supplementary Table S7).

### 3.2. Patient Characteristics

Groups treated with Portico (N = 3001) and SAPIEN 3 (N = 17,521) differed significantly in terms of patients’ age (*p* < 0.0001), heart failure status: NYHA class III/IV (*p* = 0.0002), EuroSCORE risk profile (*p* = 0.010) and STS-PROM risk profile (*p* = 0.0001). Patients in the Portico group were older (81.8 ± 4.8 vs. 80.7 ± 7.8), more frequently presented with NYHA class III/IV symptoms (72.3% vs. 66.7%) and had a higher risk profile (7.2 ± 9.5 vs. 6.4 ± 7.1 and 5.8 ± 3.6 vs. 5.3 ± 5.5 for EuroSCORE and STS PROM, respectively). There were no significant differences between the groups in terms of sex (*p* = 0.965) or BMI (*p* = 0.241).

Baseline aortic valve parameters, such as native annulus diameter and mean transaortic gradient were also significantly different. The aortic valve annulus diameter of the Portico recipients was smaller (23.4 ± 2.6 mm vs. 24.6 ± 3.4 mm, *p* < 0.0001). The initial mean transaortic gradient was lower in the Portico compared to SAPIEN 3 group (43.1 ± 16.6 mmHg vs. 44.6 ± 14.8 mmHg, *p* < 0.0001), although the effective orifice area was comparable (0.69 ± 0.19 cm^2^ vs. 0.66 ± 0.15 cm^2^, *p* = 0.352).

Patients treated with Portico received larger prostheses. The mean size of the implanted valve was 26.55 ± 1.92 mm vs. 25.10 ± 1.80 mm (*p* < 0.0001).

Transfemoral TAVI was performed in 94.9% of Portico and 94.0% of SAPIEN 3 cases.

### 3.3. Outcomes

#### 3.3.1. Procedural Outcomes and Complications

**Figure 2:**
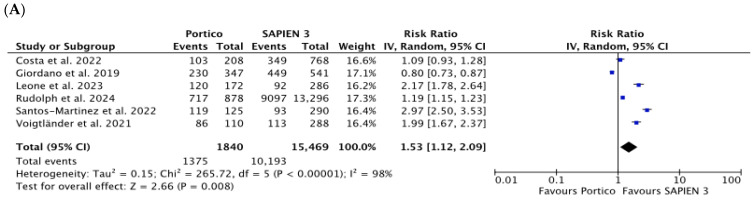

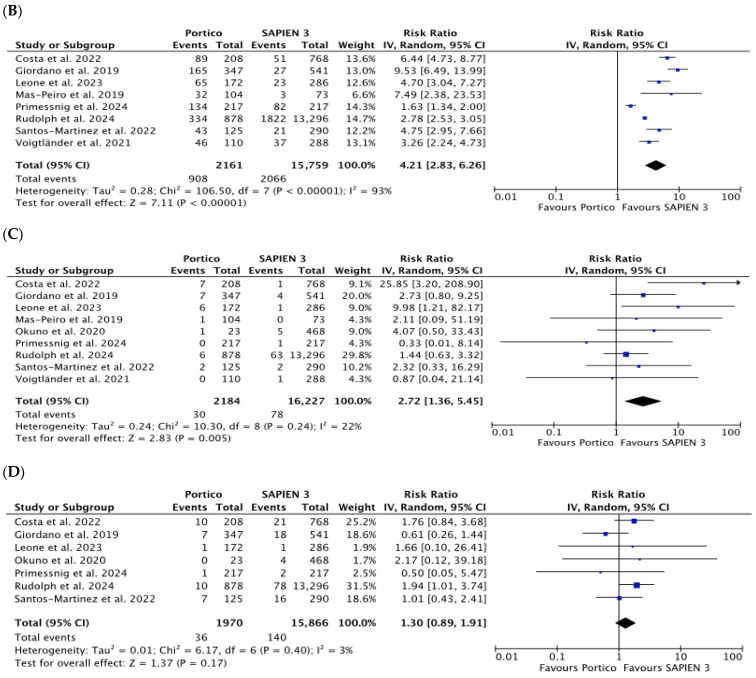
Risk ratio (RR) with corresponding 95% Confidence Intervals (CIs) for the comparison of Portico vs. SAPIEN 3 devices in the analysis of procedural outcomes: pre-dilatation (**A**), post-dilatation (**B**), need for more than one prosthesis (**C**), and other TAVI-related complications (**D**). Blue squares are point estimates for single studies; black diamond is an overall estimate.

Pre-dilatation (RR 1.53 [1.12, 2.09] *p* = 0.008; *I*^2^ = 98%) and post-dilatation (RR 4.21 [2.83, 6.26] *p* < 0.00001; *I*^2^ = 93%) were performed more frequently in the Portico group (74.7% vs. 65.9% and 42.0% vs. 13.1% for pre-dilatation and post-dilatation, respectively) (Figure 2A,B). Data were obtained from six and eight studies comprising 17,309 and 17,920 patients, respectively.

The need for more than one prosthesis during initial implantation was significantly higher among Portico recipients: 1.4% (30 of 2184) vs. 0.5% (78 of 16,277) of cases (RR 2.72 [1.36, 5.45], *p* = 0.005; *I*^2^ = 22%). Nine studies comprising 18,411 participants provided data for analysis (Figure 2C).

Seven studies comprising 17,836 patients contributed to the analysis of pooled other TAVI-related complications. There was no significant difference between the groups (RR 1.30 [0.89, 1.91], *p* = 0.17; *I*^2^ = 3%), although the event rate in the Portico group was twofold higher than in the SAPIEN 3 cohort (1.8% [36 of 1970] and 0.9% [140 of 15,866], respectively) (Figure 2D).

#### 3.3.2. Functional Outcomes

**Figure 3:**
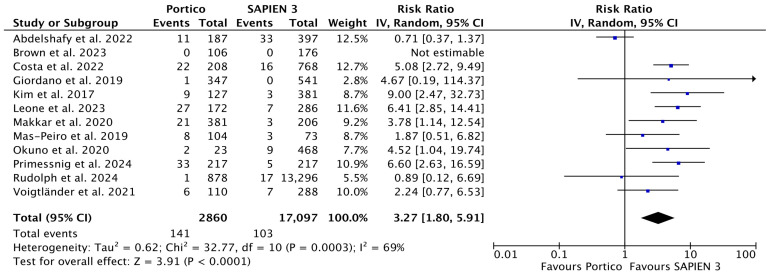
Risk Ratios (RRs) and corresponding 95% Confidence Intervals (CIs) for the comparison of Portico and SAPIEN 3 devices: moderate-to-severe PVL. Each square represents a study point estimate. Diamonds reflect the overall effect. IV, inverse variance. Blue squares are point estimates for single studies; black diamond is an overall estimate.

Twelve (N = 19,957) and five (N = 17,056) studies were included in the analysis of moderate-to-severe and mild paravalvular leak (PVL). The incidence of moderate-to-severe PVL was significantly higher in the Portico compared to SAPIEN 3 group, with corresponding rates of 4.9% (141 of 2860) and 0.6% (103 of 17,097) (RR 3.27 [1.80, 5.91], *p* < 0.0001; *I*^2^ = 69%) (Figure 3).

**Figure 4:**
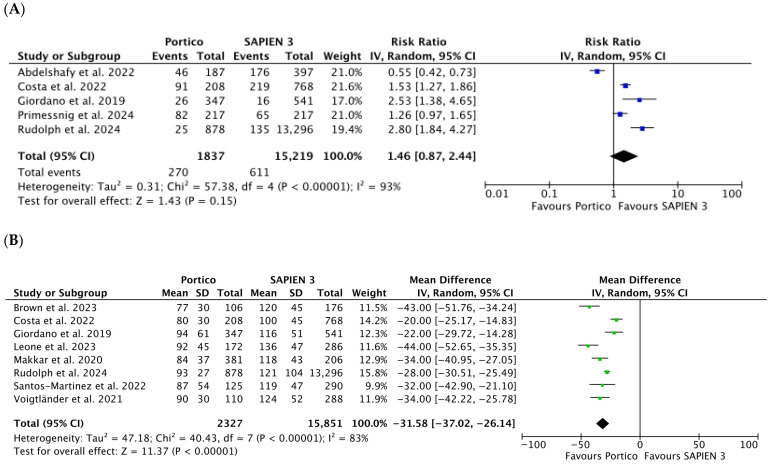

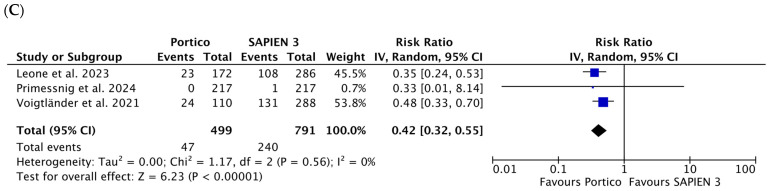
Risk Ratios s(RR) with corresponding 95% Confidence Intervals (CIs) for the comparison of Portico vs. SAPIEN 3 devices in the analysis of functional outcomes: mild PVL (**A**); Mean Difference (MD) and corresponding 95% Confidence Interval (CI) for the comparison of Portico vs. SAPIEN 3 devices in the analysis of functional outcomes: mean trans-prosthetic gradient (**B**); Risk Ratio (RR) with corresponding 95% Confidence Intervals (CIs) for the comparison of Portico vs. SAPIEN 3 devices in the analysis of functional outcomes: prosthesis–patient mismatch (**C**). Each square represents a study point estimate. Diamonds reflect the overall effect. IV, inverse variance. Blue squares are point estimates for single studies; black diamond is an overall estimate.

No significant difference in the risk of mild PVL was observed (RR 1.46 [0.87, 2.44], *p* = 0.15; *I*^2^ = 93%) despite a marked difference in event rates (14.8% [270 of 1837] and 4.0% [611 of 15,219] for Portico and SAPIEN 3, respectively) (Figure 4A).

The mean trans-prosthetic gradient and the rate of patient–prosthesis mismatch (PPM) were significantly lower in the Portico group compared with the SAPIEN 3 cohort (MD −31.58 [−37.02; −26.14] mmHg; *p* < 0.00001 and RR 0.42 [0.32, 0.55], *p* < 0.00001; *I*^2^ = 0%, respectively), whereas the prosthetic valve area was comparable (MD 7.08 [−12.14, 26.30] mm^2^; *p* = 0.47) (Figure 4B,C, Supplementary Figure S1). Data were retrieved from eight, three, and five studies comprising 18,268, 1290, and 2746 patients, respectively.

#### 3.3.3. Clinical Outcomes

- Thirty-day outcomes

**Figure 5:**
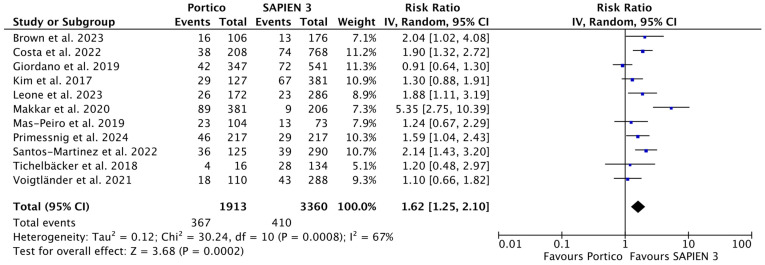
Risk Ratios (RRs) and corresponding 95% Confidence Intervals (CIs) for the comparison of Portico and SAPIEN 3 devices: PPI. Each square represents a study point estimate. Diamonds reflect the overall effect. IV, inverse variance. Blue squares are point estimates for single studies; black diamond is an overall estimate.

Patients treated with the Portico device demonstrated more than 60% higher risk of permanent pacemaker implantation (PPI) compared with those treated with SAPIEN 3 (RR 1.62 [1.25, 2.10], *p* = 0.0002; *I*^2^ = 66%). The corresponding rates were 19.0% (367 of 1913) and 12.2% (410 of 3360). Data on PPI were available from eleven studies comprising 5273 patients (Figure 5).

**Figure 6:**
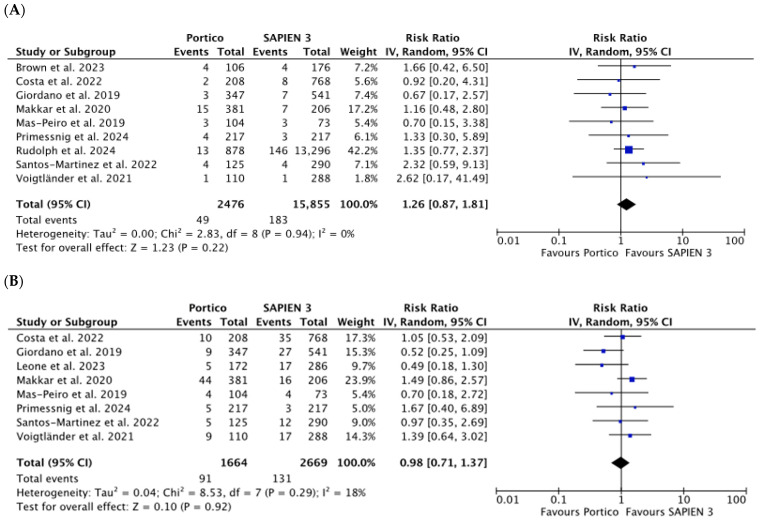
Risk Ratios (RRs) with corresponding 95% Confidence Intervals (CIs) for the comparison between Portico and SAPIEN 3 devices in the analysis of clinical outcomes: neurologic events (**A**) and life-threatening and major bleeding (**B**). Blue squares are point estimates for single studies; black diamond is an overall estimate.

No significant differences were observed between the valves with respect to neurologic events (stroke and/or TIA) (RR 1.26 [0.87, 1.81], *p* = 0.22; *I*^2^ = 0%), life-threatening and major bleeding (RR 0.98 [0.71, 1.37], *p* = 0.92; *I*^2^ = 18%) (Figure 6A,B), or other clinical endpoints, including major vascular complication (MVC) (RR 1.03 [0.71, 1.49], *p* = 0.88; *I*^2^ = 60%), AKI (RR 1.14 [0.70, 1.85], *p* = 0.60; *I*^2^ = 54%), MI (RR 1.36 [0.58, 3.17], *p* = 0.48; *I*^2^ = 0%), and mortality (RR 1.21 [0.83, 1.76], *p* = 0.32; *I*^2^ = 10%) (Supplementary Figure S2A–D).

- One-year outcomes

**Figure 7:**
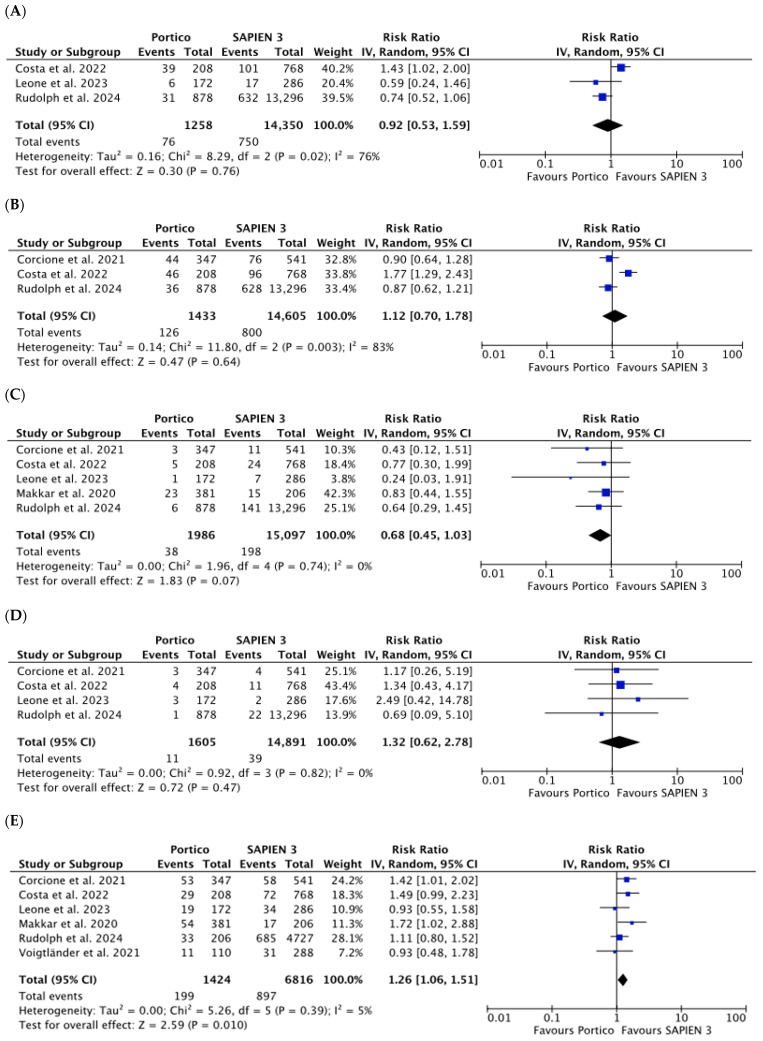
Risk Ratios (RRs) with corresponding 95% Confidence Intervals (CIs) for the comparison between Portico and SAPIEN 3 devices in the analysis of clinical outcomes at 1-year follow-up: HF rehospitalization (**A**), permanent pacemaker implantation (**B**), neurologic events (**C**), myocardial infarction (**D**) and mortality (**E**). Blue squares are point estimates for single studies; black diamond is an overall estimate.

No significant differences were noticed in HF rehospitalization (RR 0.92 [0.53, 1.59], *p* = 0.76; *I*^2^ = 76%), PPI (RR 1.12 [0.70, 1.78], *p* = 0.64; *I*^2^ = 83%), neurologic events (RR 0.68 [0.45, 1.03], *p* = 0.07; *I*^2^ = 0%), and MI (RR 1.32 [0.62, 2.78], *p* = 0.47; *I*^2^ = 0%). Data were obtained from three, three, five, and four studies including 15,608, 16,038, 17,083, and 16,496 participants, respectively (Figure 7A–D).

Six studies reported 1-year all-cause mortality. Overall, 1096 (13.3%) patients died, with corresponding rates of 14.0% (199 of 1424) and 13.2% (897 of 6816) in the Portico and SAPIEN 3 groups, respectively. A statistically significant difference was observed (RR 1.26 [1.06, 1.51], *p* = 0.01; *I*^2^ = 5%) (Figure 7E).

### 3.4. Sensitivity Analysis and Heterogeneity

Excluding individual studies sequentially and repeating the calculations for the need for more than one prosthesis, moderate-to-severe PVL, mean trans-prosthetic gradient, PPM and PPI did not change the results (Supplementary Table S8). Similarly, applying the risk difference (RD) analysis to studies reporting zero events in both arms did not alter the estimates (Supplementary Figure S3).

Substantial heterogeneity (Higgin’s *I*^2^ > 50%) was noticed for pre- and post-dilatation, PVL, major vascular complications, PPI and AKI, as well as rehospitalization and PPI, at 1-year follow-up. Clinical heterogeneity most likely arises from an imbalance between the populations being compared, whereas heterogeneity in echocardiographic outcomes likely reflects the inherent variability in the assessment of these parameters.

## 4. Discussion

To the best of our knowledge, the current study represents the only meta-analysis comparing procedural, clinical, and functional outcomes of Abbott’s Portico and Edwards’ SAPIEN 3 devices. By pooling data from one randomized trial and thirteen observational studies (including two propensity score-matched and two propensity score-weighted studies), we were able to evaluate both short- and long-term performance of the two devices in a large cohort of over 20,500 patients in a comprehensive and systematic manner.

Despite the observed imbalance in age, heart failure condition, and risk profile between the study groups, individual studies did not demonstrate marked differences between Portico and SAPIEN 3 recipients. Patients who received the Portico device were older, more frequently presented with NYHA class III/IV symptoms, and had a higher risk profile. They also had a smaller native annulus diameter and a higher mean transvalvular gradient; however, they received significantly larger valve prostheses.

The main finding of the present report was that moderate-to-severe PVL was more common in the Portico group. On the other hand, Portico was associated with a lower mean transprosthetic gradient and incidence of patient–prosthesis mismatch (PPM). The Portico group showed higher rates of at least second prosthesis utilization during initial implantation, pre- and post-dilatation, permanent pacemaker implantation (PPI), and 1-year mortality. Other TAVI-related complications, as well as 30-day and 1-year clinical outcomes, including life-threatening/major bleeding, neurologic events (stroke and/or TIA), acute kidney injury (AKI), myocardial infarction (MI) and rehospitalization due to heart failure, did not differ significantly between the devices.

We observed a statistically significant difference in the occurrence of moderate-to-severe PVL between the self-expanding Portico and the balloon-expandable SAPIEN 3 at 30-day follow-up. In the Portico group, the rate of moderate-to-severe PVL was 4.9%. Although the difference in mild PVL event rates was notable (14.8% vs. 4.0% for Portico and SAPIEN 3, respectively), it did not reach statistical significance. In the multicenter single-arm study comprising 1001 patients who received the Portico device, conducted by Möllmann et al. [27], the incidence of 30-day moderate-to-severe PVL was 2.1% (16 of 771), whereas mild PVL was observed in 53.2% of cases (410 of 771). Moderate-to-severe PVL is associated with higher long-term mortality [28,29]. Other long-term follow-up data have suggested that even mild PVL increases late mortality after implantation of the self-expanding CoreValve [30]. Ando et al. [31], in a meta-analysis comprising 21,018 patients, demonstrated higher all-cause mortality in patients with mild PVL compared to those with none/trivial PVL (RR 1.26, [1.11–1.43], *p* < 0.001]. Yokoyama et al. [32] revealed, in a matched cohort, that patients with mild PVL had a significant 1.4-fold increased risk of mortality 5 years after TAVI. A meta-analytical comparison of ACURATE neo (another self-expanding prosthesis) with SAPIEN 3 showed significanly higher 30-day incidence of moderate-to-severe and mild PVL in the ACURATE neo group, which was associated with worse survival observed at 1-year follow-up [33].

Several potential causes of PVL include severe native valve calcification, suboptimal artificial valve sizing, positioning, and deployment, as well as prosthesis design. Oh et al. reported that 83% of CoreValve recipients expirienced at least one degree of regression in PVL during one year of follow-up [34]. Popma et al. demonstrated that the frequency of moderate or severe PVL was more than 50% lower at 12 months after CoreValve TAVI (4.2%) compared to discharge (10.7%) [35]. The authors suspected that the structural properties of the nitinol self-expanding frame and its progressive expansion could improve paravalvular sealing. However, the lack of reported functional outcome data at the 1-year follow-up in assessed studies does not allow confirmation of this phenomenon. A decreasing rate of PVL with Medtronic’s self-expanding transcatheter valves was observed with each subsequent generation. The CoreValve, the precursor of the Evolut R, demonstrated nearly 50% higher risk for moderate-to-severe PVL [36]. The Evolut PRO, created by adding an external pericardial skirt to the lower part of the Evolut R frame, achieved a further reduction of nearly 35% in moderate-to-severe PVL [37]. Future studies with the Navitor, the next iteration of the Portico prosthesis equipped with an active-sealing cuff at the lower part of the frame, will determine whether a similar trend is observed with Abbott’s devices. However, comparative data for Navitor remain unavailable at this time. The ongoing ENVISION randomized clinical trial (NCT05932615) is currently evaluating the Navitor system in patients with severe symptomatic aortic stenosis deemed at low or intermediate surgical risk.

In our study, the Portico valve demonstrates very favorable short-term hemodynamic parameters with a significantly lower mean gradient and rate of PPM compared to SAPIEN 3. Patients with potential severe PPM are at great risk of mortality and kidney injury. Zorn et al. [38] reported that the rates of all-cause mortality and acute kidney injury at 1 year were significantly higher in patients with severe PPM compared to those without severe PPM (20.6% vs. 12.0% [*p* = 0.01] for mortality and 19.2% vs. 8.5% [*p* = 0.0008] for kidney injury).

Data regarding early functional results across multiple studies generally reported better hemodynamic performance of self-expanding prostheses, whereas balloon-expandable valves were associated with a lower rate of PVL, suggesting a potential class effect. To date, only one prosthetic valve, the Lotus (Boston Scientific Corporation, Marlborough, MA, USA), has demonstrated a lower rate of 30-day PVL compared to the balloon-expandable SAPIEN 3. This mechanically expanded valve showed a 35% reduction in the risk of mild PVL compared to SAPIEN 3 [39].

Our study revealed that the Portico group had higher rates of at least second prosthesis utilization, pre-dilatation, post-dilatation and PPI. The need for more than one valve during initial implantation is most likely due to the less frequent use of this prosthesis and, consequently, the operators’ limited experience. Post-dilatation may improve the hemodynamic performance of the implanted prosthesis and reduce the grade or severity of PVL. However, crushing and compressing severely calcified native tissues can damage the cardiac conduction system and/or release calcified fragments, potentially leading to stroke. In our study, pre- and post-dilatation were performed significantly more frequently in the Portico group (74.7% vs. 65.9% for pre-dilatation and 42.0% vs. 13.1% for post-dilatation, respectively). Although no difference in neurologic events (stroke and/or TIA) was observed between the devices, the rate of PPI in the Portico group was more than 60% higher than in the SAPIEN 3 group, with corresponding rates of 19.0% vs. 12.2%, respectively. In the study by Möllmann et al. [27], including 1001 Portico recipients, the rates of pre-dilatation, post-dilatation, and PPI were 86.9%, 37.6%, and 17.1%, respectively. Data from The Netherlands Heart Registration [40] showed that the incidence of PPI within 30 days following TAVI was 12%, and they identified post-dilatation as an independent risk factor.

On the other hand, Sanz Sanchez et al. [41] did not observe a higher rate of stroke and/or PPI after post-dilatation in the self-expanding valve group, consistent with the findings of Nara and colleagues in the case of balloon-expandable prostheses [42].

Several reports have highlighted a correlation between the atrioventricular conduction block and depth of prosthesis implantation, emphasizing the need for high placement of the self-expanding valves [43,44].

Two approaches may be important for reducing the rate of PPI in self-expanding prostheses: (1) avoidance of post-dilatation, based on the structural properties of the nitinol frame that allow progressive expansion to improve sealing [34,35], and (2) high placement of the valves [45]. Beyond device-specific factors, implantation technique plays a critical role in reducing conduction abnormalities. The cusp-overlap technique, as opposed to the traditional co-planar view, has been shown to facilitate the deeper implantation of self-expanding valves and reduce the rate of permanent pacemaker implantation [46]. Several studies have demonstrated that routine use of the cusp-overlap view allows for more accurate assessment of implantation depth, particularly in patients with a horizontal aorta or heavy calcification. Adoption of this technique may mitigate the higher PPI rates observed with self-expanding prostheses, including the Portico valve.

Several limitations should be acknowledged. Firstly, the present investigation consists predominantly of observational studies, which inevitably increases the risk of bias. Adjustment for differences in baseline patient characteristics through propensity score matching or weighting was applied in only four of the fourteen reports. In addition, other confounders, such as learning curve, operators’ experience, and decisions regarding valve type, remain and may further contribute to the risk of bias. Despite being assessed as having a moderate risk of bias, observational studies included in our meta-analysis appear to provide robust evidence on the topics of interest. Secondly, findings regarding clinical outcomes, particularly mortality, should be interpreted with caution, as imbalances in age and risk profile disadvantaged the Portico population. Thirdly, only one third of the studies reported a 1-year follow-up when comparing the two platforms. Consequently, our finding of increased 1-year mortality with Portico remains a weak association rather than a proven causal link, particularly given the absence of longer-term follow-up data for this device. In contrast, long-term data for other self-expanding platforms (e.g., the Medtronic CoreValve) have demonstrated that moderate-to-severe PVL present at 30 days is associated with a 2.5-fold increased risk of late mortality at 5 years [47], raising the possibility that a similar hazard may exist for Portico. Whether the higher PVL rate observed with Portico translates into excess late mortality requires confirmation from future dedicated long-term comparative studies. Fourthly, substantial heterogeneity in some procedural, clinical, and functional outcomes may affect the interpretation of the results. Fifthly, the absence of some outcomes of interest in the included studies may have contributed to publication bias. The use of VARC-2, rather than the more recent VARC-3 criteria, represents a limitation of this meta-analysis; yet, only a single study reports it. Lastly, it must be acknowledged that both valves compared in this analysis have been followed by newer generations. The SAPIEN 3 platform has been succeeded by SAPIEN 3 Ultra (featuring a 40% taller external seal skirt) and SAPIEN 3 Ultra RESILIA (incorporating RESILIA^®^ tissue for enhanced durability). Similarly, the Portico valve is being progressively replaced by the Navitor valve (equipped with an active sealing cuff). Consequently, our findings are based on older-generation platforms. Nevertheless, the core design features—balloon-expandable versus self-expanding mechanisms, frame geometry, and sealing principles—are largely preserved across generations. Therefore, although the specific valves studied may not represent the absolute latest technology, many of which are still unavailable “on the shelf”, the comparative insights regarding class effects (e.g., higher PPI rates with self-expanding valves, lower PVL rates with balloon-expandable valves, and superior hemodynamics with certain self-expanding designs) remain relevant for interpreting the performance of contemporary and future devices.

## 5. Conclusions

The findings of this study demonstrate favorable outcomes for both the Portico and SAPIEN 3 prostheses. The Portico device was associated with a significantly higher rate of moderate-to-severe paravalvular leak, as well as permanent pacemaker implantation. In contrast, the SAPIEN 3 valve was associated with a significantly higher mean trans-prosthetic gradient, with a corresponding increase in the rate of patient–prosthesis mismatch. Whether the higher paravalvular leak rate in Portico recipients accounts for the increased 1-year mortality remains to be proven in a dedicated long-term study.

## Figures and Tables

**Table 1:** Summary of the valve characteristics.

Portico	SAPIEN 3
Valve features	
Intra-annular three leaflet position	Intra-annular three leaflet position
Bovine pericardial leaflet tissue	Bovine pericardial leaflet tissue
Self-expanding nitinol frame	Balloon-expandable cobalt-chromium frame
Ability to recapture, resheath and reposition	-
Nitinol frame, large and open cell geometry	Low cobalt–chromium frame, open cell geometry
Porcine pericardial tissue inner skirt at the inflow	Polyethylene terephthalate (PET) inner skirt at the inflow
-	Textured PET external seal
Transfemoral sheath size (valve size)	
18-French (23 mm and 25 mm)	14-French (20 mm, 23 mm and 26 mm)
19-French (27 mm and 29 mm)	16-French (29 mm)
Annulus size treated: 19 mm–27 mm	Annulus size treated: 16 mm–28 mm

Portico and SAPIEN 3 devices.

**Table 2:** Baseline characteristics of included studies.

Study	Design	Study Period	Intervention	Cohort	Follow-Up (Months)	VARC-2/3 Definitions	Risk of Bias
Abdelshafy M. et al. 2022 [12]	MC, RCS	could not be defined *	Portico	187	NR	no	moderate **
			SAPIEN 3	397			
Brown J.A. et al. 2023 [13]	SC, PCS	2021–2022	Portico	106	1	no	moderate **
			SAPIEN 3	176			
Corcione N. et al. 2021 [14]/ Giordano A. et al. 2019 [16]	MC, PCS	2012–2018	Portico	347	1, 12	yes	moderate **
			SAPIEN 3	541			
Costa G. et al. 2022 [15]	MC, RCS	2016–2018	Portico	208	12	no	moderate **
			SAPIEN 3	768			
Kim W-K. et al. 2017 [17]	SC, RCS	2011–2017	Portico	127	1	yes	moderate **
			SAPIEN 3	381			
Leone P.P. et al. 2023 [18]	MC, RCS	2011–2020	Portico	172	12	yes	moderate **
			SAPIEN 3	286			
Makkar R.R. et al. 2020 [19]	MC, RCT	2014, 2015–2017	Portico	381	24	yes	low ***
			SAPIEN 3	206			
Mas-Peiro S. et al. 2019 [20]	SC, RCS, PSM	2015–2017	Portico	104	1	yes	moderate **
			SAPIEN 3	73			
Okuno T. et al. 2020 [21]	SC, RCS	2007–2018	Portico	23	12	yes	moderate **
			SAPIEN 3	468			
Primessnig U. et al. 2024 [22]	SC, RCS, PSM	2018–2021	Portico	217	1	yes	moderate **
			SAPIEN 3	217			
Rudolph T.K. et al. 2024 [23]	MC, RCS, PSW	2014–2019	Portico	878	12	no	moderate **
			SAPIEN 3	13,296			
Santos-Martinez S. et al. 2022 [24]	MC, RCS	2017–2020	Portico	125	in hospital	yes	moderate **
			SAPIEN 3	290			
Tichelbäcker T. et al. 2018 [25]	SC, PCS	2009–2015	Portico	16	in hospital	no	moderate **
			SAPIEN 3	134			
Voigtländer L. et al. 2021 [26]	MC, RCS	2012–2019	Portico	110	12	yes	moderate **
			SAPIEN 3	288			

SC, single center; MC, multicenter; RCS, retrospective cases series; PCS, prospective cohort study; RCT, randomized clinical trial; PSM, propensity score matched; PSW, propensity score weighted; VARC-2/3, Valve Academic Research Consortium–2/3; RoB2, risk-of-bias tool 2; ROBINS-I, risk of bias in non-randomized studies of intervention; NR, not reported; * study period is reported by center and not by valve; **, ROBINS-I; ***, RoB2.

**Table 3:** Baseline patient and procedural characteristics.

Study		Age	Females (%)	BMI (kg/m2)	NYHA III/IV (%)	STS-PROM (%)	EuroSCORE II (%)	Mean Gradient (mmHg)	Valve Area (cm^2^)	Annulus Diameter (mm)	Femoral Access (%)
Abdelshafy M. et al. 2022 [12]	P	NR	NR	NR	NR	NR	NR	NR	NR	NR	NR
	S3										
Brown J.A. et al. 2023 [13]	P	81.0 ± 7.5	53.8	29.2 ± 6.4	NR	NR	NR	42.3 ± 8.3	NR	24.2 ± 2.4	88.7
	S3	79.0 ± 7.5	36.4	28.4 ± 5.8				43.7 ± 15.7		25.6 ± 2.5	97.7
Corcione N. et al. 2021 [14]/Giordano A. et al. 2019 [16]	P	82.5 ± 6.5	64.3	26.8 ± 4.6	74.3	6.3 ± 4.2	4.2 ± 3.9	48.0 ± 16.8	0.69 ± 0.24	NR	87.3
	S3	83.1 ± 6.5	53.2	26.1 ± 4.6	63	5.4 ± 4.2	5.5 ± 4.8	48.2 ± 13.8	0.63 ± 0.18		94.3
Costa G. et al. 2022 [15]	P	83.1 ± 5.8	59.1	25.6 ± 4.2	75.0	NR	5.3 ± 3.4	44.3 ± 11.2	0.67 ± 0.22	NR	87.2
	S3	82.7 ± 5.2	52.0	26.0 ± 4.0	69.5		5.2 ± 3.5	46.7 ± 10.4	0.73 ± 0.07		93.6
Kim W-K. et al. 2017 [17]	P	NR	NR	NR	NR	NR	NR	NR	NR	NR	NR
	S3										
Leone P.P. et al. 2023 [18]	P	82.7 ± 5.9	91.3	26.7 ± 5.2	70.9	5.1 ± 2.7	NR	46.8 ± 15.8	0.65 ± 0.22	21.2 ± 1.3	100
	S3	82.5 ± 6.5	88.8	26.5 ± 5.5	71.0	5.7 ± 3.6		44.3 ± 15.3	0.67 ± 0.27	21.4 ± 1.0	100
Makkar R.R. et al. 2020 [19]	P	83.0 ± 7.6	51.5	NR	71.2	6.3 ± 3.4	6.6 ± 7.2	46.2 ± 11.3	0.68 ± 0.17	NR	NR
	S3	83.5 ± 7.4	51.9		73.3	6.2 ± 3.4	6.8 ± 5.9	46.7 ± 11.7	0.68 ± 0.16		
Mas-Peiro S. et al. 2019 [20]	P	81.8 ± 4.9	41.3	28.9 ± 16.5	91.4	3.9 ± 2.2	4.7 ± 3.9	NR	NR	NR	100
	S3	81.5 ± 7.3	34.2	31.4 ± 24.4	91.8	3.9 ± 2.9	5.2 ± 4.5				100
Okuno T. et al. 2020 [21]	P	NR	NR	NR	NR	NR	NR	NR	NR	NR	NR
	S3										
Primessnig U. et al. 2024 [22]	P	81.1 ± 5.2	46.1	27.7 ± 5.4	79.3	8.1 ± 13.9	16.4 ± 32.9	41.1 ± 14.4	0.77 ± 0.16	NR	100
	S3	80.0 ± 6.5	40.3	27.5 ± 5.5	71.5	10.1 ± 18.8	16.0 ± 31.4	39.9 ± 14.5	0.78 ± 0.20		100
Rudolph T.K. et al. 2024 [23]	P	80.9 ± 5.9	36.9	27.9 ± 5.3	NR	5.3 ± 4.4	NR	43.5 ± 16.9	NR	23.8 ± 3.0	100
	S3	80.5 ± 5.8	37.2	27.5 ± 5.8		5.6 ± 4.4		42.6 ± 17.3		24.7 ± 3.5	100
Santos-Martinez S. et al. 2022 [24]	P	82.6 ± 5.7	63.2	27.3 ± 4.5	43.1	NR	6.2 ± 6.1	44.8 ± 14.0	0.68 ± 0.18	23.1 ± 2.2	98.4
	S3	80.4 ± 6.4	41.4	27.4 ± 4.8	46.9		4.4 ± 4.3	45.3 ± 14.7	0.70 ± 0.17	24.8 ± 2.8	91.0
Tichelbäcker T. et al. 2018 [25]	P	NR	NR	NR	NR	NR	NR	NR	NR	NR	NR
	S3										
Voigtländer L. et al. 2021 [26]	P	83.1 ± 4.2	96.4	NR	NR	4.9 ± 2.6	NR	44.0 ± 18.0	0.37 ± 0.08 *	NR	100
	S3	82.5 ± 5.3	91.7			4.8 ± 2.5		42.3 ± 15.8	0.40 ± 0.15 *		100

P, Portico; S3, SAPIEN 3; NYHA, New York Heart Association; STS-PROM, Society of Thoracic Surgeons Predicted Risk of Mortality; EuroSCORE, European System for Cardiac Operative Risk Evaluation; NR, not reported; *, indexed.

## Data Availability

Data are available from the corresponding author upon request.

## Supplementary Materials

The following supporting information can be downloaded at: https://www.mdpi.com/article/10.3390/jcm15103573/s1, Figure S1: Forest plot: prosthetic effective orifice area (EOA); Figure S2: Forest plot: major vascular complication (A), acute kidney injury (B), peri-procedural myocardial infarction (C), 30-day mortality (D); Figure S3: RD analysis. Forest plot: moderate-to-severe PVL (A), major vascular complication (B), peri-procedural myocardial infarction (C). Table S1: MOOSE checklist; Table S2: PRISMA checklist; Table S3: Bias assessment (RoB-2, ROBINS-I); Table S4: Selection criteria for the procedure and prosthetic valve, inclusion and exclusion criteria; Table S5: Baseline patient characteristics; Table S6: Procedural characteristics; Table S7: Countries and centers; Table S8: Sensitivity analysis (leave-one-out). Reference [48] is cited in the Supplementary Materials.

## Abbreviations

TAVI	Transcatheter Aortic Valve Implantation
PPI	Permanent Pacemaker Implantation
PPM	Patient–Prosthesis Mismatch
PVL	Para-Valvular Leak
MOOSE	Meta-analysis Of Observational Studies in Epidemiology
PRISMA	Preferred Reporting Items for Systematic Reviews and Meta-Analyses
RCT	Randomized Clinical Trial
RoB2	Risk of Bias tool 2
ROBINS-I	Risk of Bias in Non-Randomized Studies of Interventions
VARC-2	Valve Academic Research Consortium-2
MVC	Major Vascular Complication
MI	Myocardial Infarction
AKI	Acute Kidney Injury
EOA	Effective Orifice Area
RR	Risk Ratio
CI	Confidence Interval
MD	Mean Difference
RD	Risk Difference
