# Supplementary material for: Outcomes of Transcatheter Aortic Valve Implantation with Abbott’s Portico Compared to Edwards’ SAPIEN 3: A Systematic Review and Meta-Analysis
Source: J Clin Med. 2026 May 7;15(10):3573. doi: 10.3390/jcm15103573 (PMC13207107; doi:10.3390/jcm15103573)

Outcomes of Transcatheter Aortic Valve Implantation with Abbott's Portico Compared to Edwards' SAPIEN 3:

A systematic Review and Meta-Analysis

SUPPLEMENTARY MATERIAL

Supplementary Table S1. MOOSE checklist

Item No	Recommendation	Reported on Page No
**Reporting of background**		
1	Problem definition	2
2	Hypothesis statement	NA
3	Description of study outcome(s)	2
4	Type of exposure or intervention used	2
5	Type of study designs used	2-3
6	Study population	3,4, 8
**Reporting of search strategy**		
7	Qualifications of searchers (eg, librarians and investigators)	3
8	Search strategy, including time period included in the synthesis and key words	2,3
9	Effort to include all available studies, including contact with authors	2,3
10	Databases and registries searched	2, Figure 1
11	Search software used, name and version, including special features used (eg, explosion)	NA
12	Use of hand searching (eg, reference lists of obtained articles)	NA
13	List of citations located and those excluded, including justification	NA
14	Method of addressing articles published in languages other than English	NA
15	Method of handling abstracts and unpublished studies	NA
16	Description of any contact with authors	NA
**Reporting of methods**		
17	Description of relevance or appropriateness of studies assembled for assessing the hypothesis to be tested	NA
18	Rationale for the selection and coding of data (eg, sound clinical principles or convenience)	NA
19	Documentation of how data were classified and coded (eg, multiple raters, blinding and interrater reliability)	NA
20	Assessment of confounding (eg, comparability of cases and controls in studies where appropriate)	2,3
21	Assessment of study quality, including blinding of quality assessors, stratification or regression on possible predictors of study results	Supplementary Table 2
22	Assessment of heterogeneity	3
23	Description of statistical methods (eg, complete description of fixed or random effects models, justification of whether the chosen models account for predictors of study results, dose-response models, or cumulative meta-analysis) in sufficient detail to be replicated	3
24	Provision of appropriate tables and graphics	yes
**Reporting of results**		
25	Graphic summarizing individual study estimates and overall estimate	NA
26	Table giving descriptive information for each study included	Table 2

Item No	Recommendation	Reported on Page No
**Reporting of discussion**		
29	Quantitative assessment of bias (eg, publication bias)	NA
30	Justification for exclusion (eg, exclusion of non-English language citations)	Figure 1
31	Assessment of quality of included studies	Supplementary Table 2
**Reporting of conclusions**		
32	Consideration of alternative explanations for observed results	17,18
33	Generalization of the conclusions (ie, appropriate for the data presented and within the domain of the literature review)	17,18
34	Guidelines for future research	17
35	Disclosure of funding source	18

From: Stroup DF, Berlin JA, Morton SC, et al for the Meta-analysis Of Observational Studies in Epidemiology (MOOSE) Group. Meta-analysis of Observational Studies in Epidemiology. A Proposal for Reporting. JAMA 2000;283:2008-2012.

Supplementary Table S2. PRISMA checklist

Section and Topic	Item #	Checklist item	Location where item is reported
**TITLE**			
Title	1	Identify the report as a systematic review.	page 1
**ABSTRACT**			
Abstract	2	See the PRISMA 2020 for Abstracts checklist.	No
**INTRODUCTION**			
Rationale	3	Describe the rationale for the review in the context of existing knowledge.	page 2, line 66-69
Objectives	4	Provide an explicit statement of the objective(s) or question(s) the review addresses.	page 2, line 71-73
**METHODS**			
Eligibility criteria	5	Specify the inclusion and exclusion criteria for the review and how studies were grouped for the syntheses.	page 3, line 83-87
Information sources	6	Specify all databases, registers, websites, organisations, reference lists and other sources searched or consulted to identify studies. Specify the date when each source was last searched or consulted.	page 3, line 78
Search strategy	7	Present the full search strategies for all databases, registers and websites, including any filters and limits used.	page 4, Figure 1
Selection process	8	Specify the methods used to decide whether a study met the inclusion criteria of the review, including how many reviewers screened each record and each report retrieved, whether they worked independently, and if applicable, details of automation tools used in the process.	page 2, line 83-87
Data collection process	9	Specify the methods used to collect data from reports, including how many reviewers collected data from each report, whether they worked independently, any processes for obtaining or confirming data from study investigators, and if applicable, details of automation tools used in the process.	Page 2
Data items	10a	List and define all outcomes for which data were sought. Specify whether all results that were compatible with each outcome domain in each study were sought (e.g. for all measures, time points, analyses), and if not, the methods used to decide which results to collect.	page 3, line 97-111
	10b	List and define all other variables for which data were sought (e.g. participant and intervention characteristics, funding sources). Describe any assumptions made about any missing or unclear information.	not applicable
Study risk of bias assessment	11	Specify the methods used to assess risk of bias in the included studies, including details of the tool(s) used, how many reviewers assessed each study and whether they worked independently, and if applicable, details of automation tools used in the process.	page 3, line 91-95
Effect measures	12	Specify for each outcome the effect measure(s) (e.g. risk ratio, mean difference) used in the synthesis or presentation of results.	page 3, line 113-114

Section and Topic	Item #	Checklist item	Location where item is reported
Synthesis methods	13a	Describe the processes used to decide which studies were eligible for each synthesis (e.g. tabulating the study intervention characteristics and comparing against the planned groups for each synthesis (item #5)).	NA
	13b	Describe any methods required to prepare the data for presentation or synthesis, such as handling of missing summary statistics, or data conversions.	page 3, line 124-126
	13c	Describe any methods used to tabulate or visually display results of individual studies and syntheses.	Figures and Supplementary Figures
	13d	Describe any methods used to synthesize results and provide a rationale for the choice(s). If meta-analysis was performed, describe the model(s), method(s) to identify the presence and extent of statistical heterogeneity, and software package(s) used.	page 3, line 113-130
	13e	Describe any methods used to explore possible causes of heterogeneity among study results (e.g. subgroup analysis, meta-regression).	page 11, line 277-281
	13f	Describe any sensitivity analyses conducted to assess robustness of the synthesized results.	page 11, line 272-276
Reporting bias assessment	14	Describe any methods used to assess risk of bias due to missing results in a synthesis (arising from reporting biases).	NA
Certainty assessment	15	Describe any methods used to assess certainty (or confidence) in the body of evidence for an outcome.	Page 13,15
**RESULTS**			
Study selection	16a	Describe the results of the search and selection process, from the number of records identified in the search to the number of studies included in the review, ideally using a flow diagram.	page 4, Figure 1
	16b	Cite studies that might appear to meet the inclusion criteria, but which were excluded, and explain why they were excluded.	page 4, Figure 1
Study characteristics	17	Cite each included study and present its characteristics.	page 5-6, Table 2
Risk of bias in studies	18	Present assessments of risk of bias for each included study.	Supplementary Table 2
Results of individual studies	19	For all outcomes, present, for each study: (a) summary statistics for each group (where appropriate) and (b) an effect estimate and its precision (e.g. confidence/credible interval), ideally using structured tables or plots.	Figures 2-7
Results of syntheses	20a	For each synthesis, briefly summarise the characteristics and risk of bias among contributing studies.	NR
	20b	Present results of all statistical syntheses conducted. If meta-analysis was done, present for each the summary estimate and its precision	Figures and

Section and Topic	Item #	Checklist item	Location where item is reported
		(e.g. confidence/credible interval) and measures of statistical heterogeneity. If comparing groups, describe the direction of the effect.	Supplementary Figures
	20c	Present results of all investigations of possible causes of heterogeneity among study results.	page 11, line 279-281
	20d	Present results of all sensitivity analyses conducted to assess the robustness of the synthesized results.	Supplementary Figures
Reporting biases	21	Present assessments of risk of bias due to missing results (arising from reporting biases) for each synthesis assessed.	NR
Certainty of evidence	22	Present assessments of certainty (or confidence) in the body of evidence for each outcome assessed.	NR
**DISCUSSION**			
Discussion	23a	Provide a general interpretation of the results in the context of other evidence.	page 15-17
	23b	Discuss any limitations of the evidence included in the review.	page 13-14, line 381-396
	23c	Discuss any limitations of the review processes used.	Page 17-18
	23d	Discuss implications of the results for practice, policy, and future research.	Page 16-17
**OTHER INFORMATION**			
Registration and protocol	24a	Provide registration information for the review, including register name and registration number, or state that the review was not registered.	Not registered
	24b	Indicate where the review protocol can be accessed, or state that a protocol was not prepared.	Not prepared
	24c	Describe and explain any amendments to information provided at registration or in the protocol.	NA
Support	25	Describe sources of financial or non-financial support for the review, and the role of the funders or sponsors in the review.	Page 18
Competing interests	26	Declare any competing interests of review authors.	Page 18
Availability of data, code and other materials	27	Report which of the following are publicly available and where they can be found: template data collection forms; data extracted from included studies; data used for all analyses; analytic code; any other materials used in the review.	Page 18

From: Page MJ, McKenzie JE, Bossuyt PM, Boutron I, Hoffmann TC, Mulrow CD, et al. The PRISMA 2020 statement: an updated guideline for reporting systematic reviews. BMJ 2021;372:n71. doi: 10.1136/bmj.n71

For more information, visit: http://www.prisma-statement.org/

Supplementary Table S3. Bias assessment (RoB-2, ROBINS-I)

**Study (randomized)**	**Bias arising from the randomization process**	**Bias due to deviations from intended interventions**	**Bias due to missing data**	**Bias in measurement of outcomes**	**Bias in selection of reported result**	**Overall bias**
Makkar R.R. et al. 2020 [19]	low	some concerns	low	low	low	LOW

**Study (PS matched)**	**Bias due to confounding**	**Bias in selection of participants into the study**	**Bias in measurement of interventions**	**Bias due to departures from intended interventions**	**Bias due to missing data**	**Bias in measurement of outcomes**	**Bias in selection of reported result**	**Overall bias**
Mas-Peiro S. et al. 2019 [20]	critical	moderate	low	low	low	serious	low	MODERATE
Primessnig U. et al. 2024 [22]	critical	moderate	low	low	low	serious	low	MODERATE

**Study (PS weighted)**	**Bias due to confounding**	**Bias in selection of participants into the study**	**Bias in measurement of interventions**	**Bias due to departures from intended interventions**	**Bias due to missing data**	**Bias in measurement of outcomes**	**Bias in selection of reported result**	**Overall bias**
Costa G. et al. 2022 [15]	critical	moderate	low	low	moderate	serious	moderate	MODERATE
Rudolph T.K. et al. 2024 [23]	critical	moderate	low	low	moderate	serious	low	MODERATE

**Study (unmatched)**	**Bias due to confounding**	**Bias in selection of participants into the study**	**Bias in measurement of interventions**	**Bias due to departures from intended interventions**	**Bias due to missing data**	**Bias in measurement of outcomes**	**Bias in selection of reported result**	**Overall bias**
Abdelshafy M. et al. 2022 [12]	critical	critical	moderate	low	low	low	low	MODERATE

Brown J.A. et al. 2023 [13]	critical	critical	low	low	low	serious	moderate	MODERATE
Corcione N. et al. 2021 [14] / Giordano A. Et al. 2019 [16]	critical	critical	low	low	low	serious	low	MODERATE
Kim W-K. et al. 2017 [17]	critical	critical	low	low	moderate	serious	low	MODERATE
Leone P.P. et al. 2023 [18]	critical	critical	low	low	low	serious	low	MODERATE
Okuno T. et al. 2020 [21]	critical	critical	low	low	low	serious	moderate	MODERATE
Santos-Martinez S. et al. 2022 [24]	critical	critical	low	low	low	low	low	LOW/MODERATE
Tichelbäcker T. et al. 2018 [25]	critical	critical	low	low	low	serious	serious	MODERATE/SERIOUS
Voigtländer L. et al. 2021 [26]	critical	critical	low	low	low	serious	low	MODERATE

Supplementary Table S4. Selection criteria for the procedure and prosthetic valve, inclusion and exclusion criteria.

Study	Selection criteria for the procedure / inclusion criteria	Exclusion criteria	Selection criteria for the prosthetic valve
Abdelshafy M. et al. 2022 [12]	„Multicentre retrospective corelab pooled analysis of aortograms from over 2,665 consecutive patients treated with 14 different THV devices."	not reported	not reported
Brown J.A. et al. 2023 [13]	„All patients who underwent a TAVI during time frame of the study were included for analysis. Patients were categorized by the type of THV implanted: Portico versus SAPIEN 3 Ultra versus Evolut PRO+."	not reported	not reported
Corcione N. et al. 2021 [14] / Giordano A. Et al. 2019 [16]	„All patients in whom TAVI was attempted at participating RISPEVA study centers and willing to provide consent were offered inclusion in the study, without any additional selection criterion. Accordingly, patient selection was at physician's discretion."	not reported	„Procedural strategy was at physician's discretion."
Costa G. et al. 2022 [15]	„We considered patients enrolled in the OBSERVANT II study receiving Medtronic Evolut R or PRO, Edwards SAPIEN 3, Boston ACURATE neo, or Abbott Portico TAV. Patients were divided in to 5 different groups according to the TAV implanted."	not reported	not reported
Kim W-K. et al. 2017 [17]	„Consecutive patients with severe native aortic stenosis who underwent transfemoral TAVI between 01.2011 and 05.2017 and for whom MDCT scans of the aortic root were available were included. All patients were discussed in an heart team, and decisions were made in adherence to existing guidelines."	not reported	„All patients were discussed in an interdisciplinary heart team, and decisions were made in adherence to existing guidelines."
Leone P.P. et al. 2023 [18]	„The retrospective TAVI-SMALL 2 registry included a total of 1,378 patients with severe aortic valve stenosis and small aortic annuli (defined as an annular area <400 mm2 and/or annular perimeter <72 mm on computed tomography) treated with transfemoral implantation of current-generation SEV (Evolut R and Evolut PRO; ACURATE neo; Portico and BEV (SAPIEN 3 at 16 high-volume centres. Inclusion criteria were implantation via	„Valve-in-valve procedures, TAVI for pure aortic regurgitation and lack of preprocedural computed tomographic data."	„Prosthesis type and size selection, as well as implantation technique and subsequent antithrombotic therapy, were left to the discretion of the treating physician at each centre."

	the transfemoral route of current-generation transcatheter heart valves in native aortic stenosis (both tricuspid and non-tricuspid anatomies) in patients with small aortic annuli. Local Heart Teams evaluated all patients and confirmed the indications for TAVI.”		
Makkar R.R. et al. 2020 [19]	„Potential participants were screened by the investigational sites’ multidisciplinary heart teams. Eligible patients were aged 21 years or older, in New York Heart Association functional class II or higher, had severe native aortic stenosis, were at high or extreme surgical risk. An aortic annulus diameter of 19–27 mm based on CT assessment was required for inclusion in the trial. Transcatheter vascular access via the iliofemoral artery or an alternative access route (subclavian or axillary, transaortic, or transapical)”	„Non-calcified, congenital unicuspid or bicuspid aortic valve. Mixed aortic valve disease. MI within 30 days prior. Any percutaneous coronary or peripheral interventional procedure performed within 30 days prior. Pre-existing prosthetic heart valve/ring or other implant in any valve position. Severe circumferential mitral annular calcification which is continuous with calcium in the LVOT. Severe (greater than 3+) mitral insufficiency, or severe stenosis with pulmonary compromise. Leukopenia, acute anemia, thrombocytopenia. History of bleeding diathesis or coagulopathy. Cardiogenic shock. Untreated clinically significant coronary artery disease requiring revascularization. Hemodynamic instability. Need for emergency surgery for any reason. HOCM. LVEF<20%. Intracardiac mass, thrombus or vegetation. Active peptic ulcer or upper GI bleeding within 3 months prior. Hypersensitivity or contraindication to aspirin, heparin, ticlopidine, or clopidogrel, or sensitivity to contrast media. Recent CVA or TIA. Renal insufficiency (creatinine > 3.0 mg/dL) and/or chronic dialysis. Life expectancy < 12. Significant aortic disease. Aortic root angulation > 70°. Currently participating in an investigational drug or device study. Active bacterial endocarditis within 6 months. Bulky calcified aortic valve leaflets in close proximity to coronary	„Eligible participants were randomly assigned (1:1) to receive either a Portico or commercially available valve. Selection of the commercially available valve was not randomly assigned but left to the discretion of the study site investigator.”

		ostia. Non-calcified aortic annulus. Severe Iliofemoral vessel obstructive calcification, or severe tortuosity."	
Mas-Peiro S. et al. 2019 [20]	„Based on the institutional heart-team decision, a TAVR was performed in 273 patients. Patients with symptomatic severe aortic stenosis (AS) who underwent transfemoral TAVR at our tertiary hospital from 03.2015 to 09.2017."	„ TAVR with transapical or through a non-transfemoral route, patients receiving other valves than Portico and SAPIEN 3, patients with an isolated aortic regurgitation and those receiving a valve-in-valve procedure."	„Valve choice was left to the discretion of the heart team or operator in most cases."
Okuno T. et al. 2020 [21]	„The present analysis included patients that underwent TAVR for native aortic valve stenosis with CE-marked devices between 08.2007 and 06.2018."	not reported	„The THV type and size as well as access route were determined by the team."
Primessnig U. et al. 2024 [22]	„In this retrospective comparative-cohort study, a total of 1,901 patients, who underwent TAVR between 01.2018 and 12.2021 at Charité University Medical Center were included. Procedures were performed based on the institutional heart team's collaborative decision."	„Patients who received valves other than the study valves or underwent non-transfemoral TAVR, as well as cases with missing data."	„Valve prosthesis selection was based on the decision of the heart team or operator."
Rudolph T.K. et al. 2024 [23]	„Data of all patients who underwent transfemoral TAVI between 2014 and 2019 were extracted from the database of GARY. Prostheses studied in this analysis included the balloon-expandable SAPIEN 3 and the self-expanding Acurate neo, Evolut R and Portico."	not reported	„Prostheses selection was at the discretion of the operating physician."
Santos-Martinez S. et al. 2022 [24]	„Retrospective consecutive registry from 9 European institutions of patients with symptomatic severe tricuspid AS between 01.2017 and 12.2020, who received Myval, Sapien-3, or any of the following 4 SE devices: Evolut R/PRO, ACURATE neo, Portico, and ALLEGRA. In all participating institutions, the Heart Team determined patient suitability and eligibility to perform TAVI."	not reported	„In all participating institutions, the Heart Team determined patient suitability and eligibility to valve type."
Tichelbäcker T. et al. 2018 [25]	„We analyzed the ECGs and clinical parameters of 494 consecutive patients treated with transfemoral TAVR from April 2009 to August 2015. Selection of the TAVR patients was performed by our local Heart Team."	„Patients with in-hospital deaths; a previously implanted pacemaker, ICD or CRT; valve-in-valve procedures; patients with already implanted devices; patients who died in the peri-interventional phase and patients with valve-in-valve procedures."	„Careful valve selection was completed individually for each patient by the Heart Team."
Voigtländer L. et al. 2021 [26]	„This retrospective analysis included transfemoral TAVI procedures performed between 2012 and 2019 with balloon-expanding Sapien-3, self-expanding Evolut-R/Pro,	„Patients with valve-in-valve procedures, treatment of predominant aortic	not reported

	Acurate-Neo, Portico) and mechanically expanding Lotus devices. Patients with a SAA (defined as annulus area < 400 mm2, measured by multidetector computed tomography (MDCT)) were included.”	regurgitation and procedures via non-transfemoral access routes.”	

HOCM, hypertrophic cardiomyopathy; LVEF, left ventricular ejection fraction; MI, myocardial infarction; CVA, cerebrovascular accident; TIA, transient ischemic attack; CE, ConformitéEuropéenn

Supplementary Tale S5. Baseline patient characteristics

Study [ref]	Intervention	HT (%)	DM (%)	PVD (%)	CKI (%)	COPD (%)	PM/ICD (%)	AF (%)	CAD (%)	MI history (%)	Stroke/CVD (%)	Heart surgery (%)	NYHA III/IV (%)	LVEF (%)	Mean aortic valve gradient [mmHg]	Aortic valve area (cm^2)	Aortic valve annulus diameter (mm)
Abdelshafy M. et al. 2022 [12]	P	NR	NR	NR	NR	NR	NR	NR	NR	NR	NR	NR	NR	NR	NR	NR	NR
	S3	NR	NR	NR	NR	NR	NR	NR	NR	NR	NR	NR	NR	NR	NR	NR	NR
Brown J.A. et al. 2023 [13]	P	NR	35.9	20.8	NR	33.0	NR	NR	NR	31.1	22.6	NR	NR	60.8±3.7	42.3±8.3	NR	24.2±2.4
	S3	NR	36.9	17.1	NR	29.0	NR	NR	NR	30.7	19.3	NR	NR	57.5±7.5	43.7±15.7	NR	25.6±2.5
Corcione N. et al. 2021 [14] / Giordano A. Et al. 2019 [16]	P	NR	NR	NR	15.8	NR	8.9	NR	NR	NR	6.3	6.3	74.3	54.0±10.0	48.0±16.8	0.69±0.24	NR
	S3	NR	NR	NR	15.2	NR	7.2	NR	NR	NR	7.4	7.4	63.0	53.0±10.0	48.2±13.8	0.63±0.18	NR
Costa G. et al. 2022 [15]	P	NR	27.5	13.7	14.0	13.7	NR	22.8	25.2	16.2	3.0	21.6	75.0	53.3±11.2	44.3±11.2	0.67±0.22	NR
	S3	NR	28.0	20.3	12.7	14.2	NR	23.8	24.7	16.2	2.4	13.6	69.5	55.0±7.4	46.7±10.4	0.73±0.07	NR
Kim W-K. et al. 2017 [17]	P	NR	NR	NR	NR	NR	NR	NR	NR	NR	NR	NR	NR	NR	NR	NR	NR
	S3	NR	NR	NR	NR	NR	NR	NR	NR	NR	NR	NR	NR	NR	NR	NR	NR
Leone P.P. et al. 2023 [18]	P	86.6	30.8	10.0	NR	6.4	11.6	32.8	36.3	10.5	12.3	5.8	70.9	59.9±9.2	46.8±15.8	0.65±0.22	21.2±1.3
	S3	89.2	26.9	9.5	NR	16.1	14.3	21.9	46.8	10.2	14.7	7.3	71.0	62.2±10.2	44.3±15.3	0.67±0.27	21.4±1.0
Makkar R.R. et al. 2020 [19]	P	94.4	37.3	18.7	25.1	41.6	14.7	32.5	69.6	14.4	16.0	22.9	71.2	57.3±11.3	46.2±11.3	0.68±0.17	NR
	S3	89.3	35.4	14.1	21.4	39.3	18.9	39.3	72.8	9.7	20.8	20.9	73.3	57.1±10.8	46.7±11.7	0.68±0.16	NR
Mas-Peiro S. et al. 2019 [20]	P	NR	39.4	24	59.6	13.5	12.5	NR	58.7	16.3	18.2	7.7	91.4	50.0±10.0	NR	NR	NR
	S3	NR	30.1	21.9	54.8	12.2	13.7	NR	58.9	12.3	19.2	8.2	91.8	50.0±12.6	NR	NR	NR
Okuno T. et al. 2020 [21]	P	NR	NR	NR	NR	NR	NR	NR	NR	NR	NR	NR	NR	NR	NR	NR	NR
	S3	NR	NR	NR	NR	NR	NR	NR	NR	NR	NR	NR	NR	NR	NR	NR	NR
Primessnig U. et al. 2024 [22]	P	88.5	35.9	NR	40.6	NR	NR	22.3	61.8	NR	NR	NR	79.3	54.6±11.1	41.1±14.4	0.77±0.16	NR
	S3	87.6	33.2	NR	32.3	NR	NR	20.0	59.0	NR	NR	NR	71.5	52.0±12.3	39.9±14.5	0.78±0.20	NR
Rudolph T.K. et al. 2024 [23]	P	91.3	35.6	18.2	49.6	14.3	NR	NR	51.5	NR	14.7	14.1	NR	54.2±12.7	43.5±16.9	NR	23.8±3.0
	S3	88.5	32.4	27.1	49.2	16.0	NR	NR	57.6	NR	15.3	12.8	NR	51.4±13.8	42.6±17.3	NR	24.7±3.5
Santos-Martinez S. et al. 2022 [24]	P	NR	35.2	5.0	26.4	4.8	13.6	23.2	30.4	NR	11.2	9.6	43.1	57.7±12.1	44.8±14.0	0.68±0.18	23.1±2.2
	S3	NR	3.0	13.4	27.2	13.8	10.3	21.9	45.2	NR	15.9	8.3	46.9	57.3±11.9	45.3±14.7	0.70±0.17	24.8±2.8
Tichelbäcker T. et al. 2018 [25]	P	NR	NR	NR	NR	NR	NR	NR	NR	NR	NR	NR	NR	NR	NR	NR	NR
	S3	NR	NR	NR	NR	NR	NR	NR	NR	NR	NR	NR	NR	NR	NR	NR	NR
Voigtländer L. et al. 2021 [26]	S	NR	33.6	NR	NR	10.9	NR	NR	50.9	NR	12.7	NR	NR	NR	44.0±18.0	0.37±0.08*	NR
	S3	NR	24.0	NR	NR	18.8	NR	NR	56.9	NR	14.9	NR	NR	NR	42.3±15.8	0.4±0.15*	NR

P, Portico; S3, SAPIEN 3; HT, hypertension; DM, diabetes mellitus; PVD, peripheral vascular disease; CKI, chronic kidney injury; COPD, chronic obstructive pulmonary disease; CVD, cerebrovascular disease; PM/ICD, pacemaker/implantable cardioverter-defibrillator; AF, atrial fibrillation; CAD, coronary artery disease; MI, miocardial infarction; LVEF, left ventricle ejection fraction; NR, not reported

Supplementary Table S6. Procedural characteristics

Study [ref]		Local anesthesia (%)	Femoral access site (%)	Valve sizes implanted (mean±SD)	Pre-dilatation (%)	Post-dilatation (%)	Contrast volume (ml)	Fluoroscopy time (minutes)	Procedure duration (minutes)
Abdelshafy M. et al. 2022 [12]	P	NR	NR	NR	NR	NR	NR	NR	NR
	S3	NR	NR	NR	NR	NR	NR	NR	NR
Brown J.A. et al. 2023 [13]	P	NR	88.7	27.1±1.8	NR	NR	NR	NR	NR
	S3	NR	97.7	26.5±2.1	NR	NR	NR	NR	NR
Corcione N. et al. 2021 [14] / Giordano A. Et al. 2019 [16]	P	90.2	87.3	26.4±2.2	66.2	47.6	NR	26.4±14.1	87.4±43.1
	S3	77.6	94.3	25.0±2.4	88.3	5.0	NR	21.5±14.8	113.1±46.6
Costa G. et al. 2022 [15]	P	80.2	93.7	NR	49.8	42.9	156.7±67.2	NR	NR
	S3	56.7	87.2	NR	45.5	6.7	165.8±79.9	NR	NR
Kim W-K. et al. 2017 [17]	P	NR	NR	NR	NR	NR	NR	NR	NR
	S3	NR	NR	NR	NR	NR	NR	NR	NR
Leone P.P. et al. 2023 [18]	P	NR	100	NR	70.0	38.2	NR	NR	NR
	S3	NR	100	NR	32.9	8.2	NR	NR	NR
Makkar R.R. et al. 2020 [19]	P	NR	NR	NR	NR	NR	NR	NR	NR
	S3	NR	NR	NR	NR	NR	NR	NR	NR
Mas-Peiro S. et al. 2019 [20]	P	NR	100	27.7±1.6	NR	NR	240.0±330.8	24.4±28.5	NR
	S3	NR	100	27.2±1.6	NR	NR	236.7±385.7	84.8±167.3	NR
Okuno T. et al. 2020 [21]	P	NR	NR	NR	NR	NR	NR	NR	NR
	S3	NR	NR	NR	NR	NR	NR	NR	NR
Primessnig U. et al. 2024 [22]	P	25.2	femoral 100	27.1±1.9	NR	61.8	127.0±51.4	13.4±6.0	82.7±34.9
	S3	21.8	femoral 100	26.3±2.5	NR	37.8	96.5±46.0	12.9±9.3	80.9±41.4
Rudolph T.K. et al. 2024 [23]	P	NR	femoral 100	NR	81.7	38.0	145.9±0.9	15.2±0.2	NR
	S3	NR	femoral 100	NR	60.9	13.7	126.0±0.9	13.2±0.2	NR
Santos-Martinez S. et al. 2022 [24]	P	NR	femoral 98.4	NR	95.2	35.8	NR	NR	NR
	S3	NR	femoral 91.0	NR	32.1	7.3	NR	NR	NR
Tichelbäcker T. et al. 2018 [25]	P	NR	NR	NR	NR	NR	NR	NR	NR
	S3	NR	NR	NR	NR	NR	NR	NR	NR
Voigtländer L. et al. 2021 [26]	P	71.3	femoral 100	24.3±1.5	78.2	41.8	145.4±75.3	NR	52.6±24.1
	S3	68.0	femoral 100	23.0±0.0	39.2	2.8	107.7±60.1	NR	54.9±35.8

NR, not reported

Supplementary Table S7. Countries and centers

Study	Country/Center
Abdelshafy M. et al. 2022 [12]	Ireland (Galway), Italy (Milan, Caserta, Monza and Brianza), Germany (Bad Soden), Sweden (Stockholm)
Brown J.A. et al. 2023 [13]	USA (Pittsburgh)
Corcione N. et al. 2021 [14] / Giordano A. Et al. 2019 [16]	Italy (Milan, Castel Volturno, Siena, Massa, Rozzano, Rome, Catania, Brescia, Pisa, Turin, Salerno, Lecco, Parma, Catanzaro, Genova, Seriate, Legnano, Trento, Ome, Brescia)
Costa G. et al. 2022 [15]	OBSERVANT II, (28 centres in Italy)*
Kim W-K. et al. 2017 [17]	Germany (Bad Nauheim, Dortmund, Giessen), The Netherlands (Amsterdam)
Leone P.P. et al. 2023 [18]	USA (New York), Japan (Kanagawa), Portugal (Lisbon), The Netherlands (Rotterdam), Switzerland (Zurich), Germany (Bad Nauheim), Italy (Brescia, Verona, Milan, Ravenna, Bologna, Catania)
Makkar R.R. et al. 2020 [19]	USA (Los Angeles, Washington, Asheville, New Brunswick, Newark, Indianapolis, Wichita, Oak Lawn, Palos Park), Australia (Adelaide, Sydney, Murdoch)
Mas-Peiro S. et al. 2019 [20]	Germany (Frankfurt am Main)
Okuno T. et al. 2020 [21]	Switzerland (Bern)
Primessnig U. et al. 2024 [22]	Germany (Berlin)
Rudolph T.K. et al. 2024 [23]	Germany (German Aortic Valve Registry)
Santos-Martinez S. et al. 2022 [24]	Spain (Valladolid, Madrid, Gran Canaria), The Netherlands (Breda), Ireland (Galway); Italy (Milan, Roma)
Tichelbäcker T. et al. 2018 [25]	Germany (Goettingen)
Voigtländer L. et al. 2021 [26]	Germany (Bad Nauheim, Bonn, Hamburg, Cologne)

*1. A.O.U. Città della Salute e della Scienza di Torino (TO)—Mauro Rinaldi, Stefano Salizzoni. 2. A.O. S. Croce e Carle (CN)—Giuseppe Musumeci, Giorgio Baralis. 3. A.O. SS. Antonio e Biagio e Cesare Arrigo (AL)—Gianfranco Pistis, Maurizio Reale. 4. I.R.C.C.S Policlinico San Donato (San Donato Milanese—MI)—Francesco Bedogni, Giovanni Bianchi. 5. I.R.C.C.S Multimedica (Sesto San Giovanni—MI)—Flavio Airoldi, Iassen Michev. 6. Fondazione I.R.C.C.S. Policlinico San Matteo (PV)—Maurizio Ferrario, Umberto Canosi. 7. ASST Lecco—Ospedale "A. Manzoni" (LC)—Luigi Piatti, Gianluca Tiberti. 8. ASST degli Spedali Civili—Presidio Ospedaliero di Brescia (BS)—Federica Ettori (retired), Salvatore Curello, Marianna

Adamo. 9. I.R.C.C.S Ospedale San Raffaele (MI)—Antonio Colombo, Matteo Montorfano, Marco Ancona. 10. ASST Monza & Brianza—Ospedale S. Gerardo (MB)—Virgilio Colombo, Ivan Calchera. 11. Fondazione Poliambulanza (BS)—Ornella Leonzi, Diego Maffeo. 12. ASST Papa Giovanni XXIII (BG)—Orazio Valsecchi, Federica Roncali, Angelina Vassileva. 13. Policlinico di Monza (MB)—Filippo Scalise, Giovanni Sorropago. 14. A.O. di Padova—Centro Gallucci (PD)—Giuseppe Tarantini, Alessandro Schiavo. 15. Hesperia Hospital (MO)—Giuseppe D’Anniballe, Davide Gabbieri. 16. A.O.U. di Parma (PR)—Luigi Vignali, Michela Bollettino. 17. A.O.U. Careggi (FI)—Carlo Di Mario, Francesco Meucci. 18. A.O.U. Senese—Ospedale Santa Maria alle Scotte (SI)—Carlo Pierli (retired), Massimo Fineschi, Alessandro Iadanza. 19. Fondazione Toscana Gabriele Monasterio—Ospedale del Cuore "G. Pasquinucci" (MS)—Sergio Berti, Giuseppa Lo Surdo. 20. Ospedale San Filippo Neri (RM)—Giulio Speciale, Andrea Bisciglia. 21. Fondazione Policlinico Universitario Agostino Gemelli IRCCS—Università Cattolica del Sacro Cuore (RM)—Carlo Trani, Diana Verdirosi. 22. A.O. San Camillo Forlanini (RM)—Roberto Violini, Laura Zappavigna. 23. A.O. San Giuseppe Moscati (AV)—Emilio Di Lorenzo, Michele Capasso. 24. A.O.U. Federico II (NA)—Giovanni Esposito, Fabio Magliulo. 25. A.O.U. OO.RR. San Giovanni di Dio e Ruggi d’Aragona (SA)—Pietro Giudice, Tiziana Attisano. 26. A.O.U.C. Policlinico di Bari (BA)—Alessandro Santo Bortone, Emanuela De Cillis. 27. A.O.U. Policlinico-Vittorio Emanuele, Università di Catania (CT)—Corrado Tamburino, Marco Barbanti. 28. Centro Cuore Morgagni—Pedara (CT)—Sebastiano Immè, Martina Patanè.

Supplementary Table S8. Sensitivity analysis - leave-one-out

Study [ref]	more than 1 prosthesis		moderate-to-severe PVL		MG		PPM		PPI	
	RR 95% CI	P	RR 95% CI	P	MD 95% CI	P	RR 95% CI	P	RR 95% CI	P
Abdelshafy M. et al. 2022 [12]			4.53 [3.24, 6.35]	0.000						
Brown J.A. et al. 2023 [13]			3.27 [1.80, 5.91]	0.000	-30.00 [-35.29, -24.72]	0.000			1.59 [1.21, 2.09]	0.000
Corcione N. et al. 2021 [14] / Giordano A. Et al. 2019 [16]	2.86 [1.19, 6.84]	0.020	3.23 [1.75, 5.96]	0.000	-32.95 [-38.89, -27.01]	0.000			1.74 [1.37, 2.21]	0.000
Costa G. et al. 2022 [15]	2.03 [1.15, 3.58]	0.010	3.07 [1.57, 6.01]	0.001	-33.44 [-38.98, -27.91]	0.000			1.59 [1.20, 2.13]	0.001
Kim W-K. et al. 2017 [17]			2.97 [1.60, 5.50]	0.000					1.67 [1.25, 2.22]	0.000
Leone P.P. et al. 2023 [18]	2.36 [1.19, 4.69]	0.010	2.99 [1.57, 5.68]	0.000	-29.83 [-34.96, -24.69]	0.000	0.48 [0.33, 0.69]	0.000	1.60 [1.21, 2.12]	0.001
Makkar R.R. et al. 2020 [19]			3.22 [1.68, 6.15]	0.000	-31.28 [-37.39, -25.17]	0.000			1.47 [1.20, 1.80]	0.000
Mas-Peiro S. et al. 2019 [20]	2.83 [1.32, 6.07]	0.008	3.44 [1.82, 6.53]	0.000					1.66 [1.26, 2.19]	0.000
Okuno T. et al. 2020 [21]	2.68 [1.23, 5.85]	0.010	3.18 [1.68, 6.00]	0.000						
Primessnig U. et al. 2024 [22]	2.94 [1.48, 5.84]	0.002	3.00 [1.58, 5.68]	0.000			0.42 [0.31, 0.56]	0.000	1.63 [1.22, 2.17]	0.000
Rudolph T.K. et al. 2024 [23]	3.58 [1.64, 7.81]	0.001	3.52 [1.91, 6.49]	0.000	-32.43 [-39.91, -24.96]	0.000				
Santos-Martinez S. et al. 2022 [24]	2.87 [1.29, 6.40]	0.010			-31.57 [-37.49, -25.64]	0.000			1.57 [1.19, 2.07]	0.001
Tichelbäcker T. et al. 2018 [25]									1.65 [1.26, 2.16]	0.000
Voigtländer L. et al. 2021 [26]	2.93 [1.39, 6.17]	0.005	3.40 [1.77, 6.54]	0.000	-31.29 [-37.31, -25.28]	0.000	0.35 [0.24, 0.53]	0.000	1.69 [1.28, 2.22]	0.000

PVL, paravalvular leak; MG, mean gradient; PPM, prosthesis-patient mismatch; PPI, permanent pacemaker implantation; RR, risk ratio; CI, confidence interval.

Supplementary figures legend:

Supplementary Figure S1: Mean Difference (MD) and corresponding 95% Confidence Interval (CI) for the comparison of Portico vs. SAPIEN 3 devices in the analysis of functional outcomes: prosthetic effective orifice area (EOA).

Supplementary Figure S2: Risk ratio (RR) with corresponding 95% Confidence Intervals (CI) for the comparison of Portico vs. SAPIEN 3 devices in the analysis of clinical outcomes: major vascular complication (A); acute kidney injury (B), peri-procedural myocardial infarction (C) and 30-day mortality (D).

Supplementary Figure S3: Risk difference (RD) analysis for endpoints including studies reporting "0" events for the comparison of Portico vs. SAPIEN 3: moderate-to-severe PVL (A), major vascular complication (B), peri-procedural myocardial infarction (C).

Supplementary Figure S1

Study or Subgroup	Portico Mean	Portico SD	Portico Total	SAPIEN 3 Mean	SAPIEN 3 SD	SAPIEN 3 Total	Weight	Mean Difference IV, Random, 95% CI
Giordano et al. 2019	104	47	347	137	47	541	20.1%	-33.00 [-39.34, -26.66]
Leone et al. 2023	97	28	172	84	19	286	20.3%	13.00 [8.27, 17.73]
Makkar et al. 2020	185	46	381	159	42	206	20.0%	26.00 [18.64, 33.36]
Santos-Martinez et al. 2022	187	54	125	167	48	290	19.3%	20.00 [9.04, 30.96]
Voigtländer et al. 2021	93	22	110	83	22	288	20.3%	10.00 [5.17, 14.83]
**Total (95% CI)**			**1135**			**1611**	**100.0%**	**7.08 [-12.14, 26.30]**

Heterogeneity: $Tau^2 = 467.35$; $Chi^2 = 191.21$, df = 4 ($P < 0.00001$); $I^2 = 98\%$
Test for overall effect: $Z = 0.72$ ($P = 0.47$)

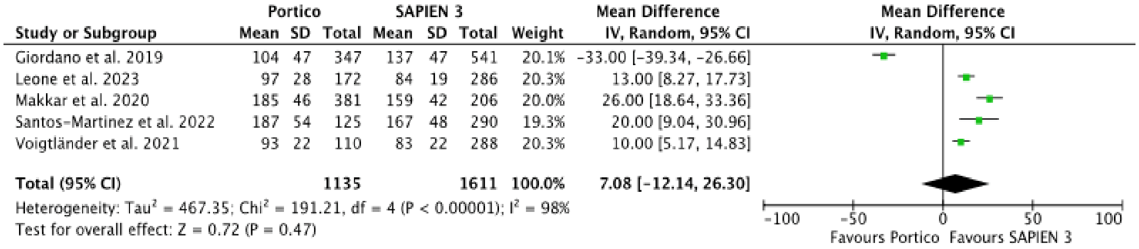

Supplementary Figure S2

A

Study or Subgroup	Portico Events	Portico Total	SAPIEN 3 Events	SAPIEN 3 Total	Weight	Risk Ratio IV, Random, 95% CI
Brown et al. 2023	0	106	0	176		Not estimable
Costa et al. 2022	9	208	25	768	11.8%	1.33 [0.63, 2.80]
Giordano et al. 2019	17	347	68	541	15.7%	0.39 [0.23, 0.65]
Leone et al. 2023	9	172	16	286	11.0%	0.94 [0.42, 2.07]
Makkar et al. 2020	36	381	15	206	14.6%	1.30 [0.73, 2.31]
Mas-Peiro et al. 2019	6	104	4	73	6.4%	1.05 [0.31, 3.60]
Primessnig et al. 2024	2	217	1	217	2.1%	2.00 [0.18, 21.89]
Rudolph et al. 2024	78	878	944	13296	21.0%	1.25 [1.00, 1.56]
Santos-Martinez et al. 2022	3	125	10	290	6.1%	0.70 [0.19, 2.49]
Voigtländer et al. 2021	10	110	14	288	11.2%	1.87 [0.86, 4.08]
**Total (95% CI)**		**2648**		**16141**	**100.0%**	**1.03 [0.71, 1.49]**
Total events	170		1097			

Heterogeneity: $Tau^2 = 0.16$; $Chi^2 = 20.19$, df = 8 (P = 0.010); $I^2 = 60\%$
Test for overall effect: Z = 0.15 (P = 0.88)

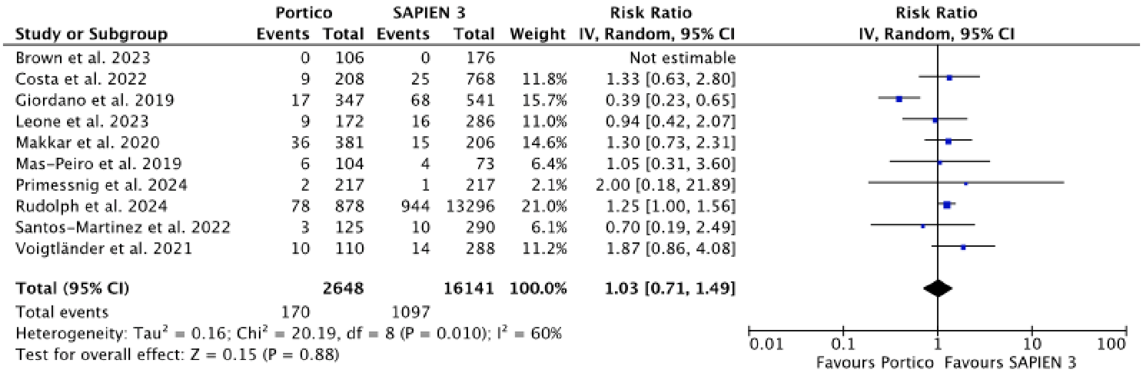

B

Study or Subgroup	Portico Events	Portico Total	SAPIEN 3 Events	SAPIEN 3 Total	Weight	Risk Ratio IV, Random, 95% CI
Costa et al. 2022	17	208	61	768	24.6%	1.03 [0.61, 1.72]
Giordano et al. 2019	16	347	47	541	23.6%	0.53 [0.31, 0.92]
Makkar et al. 2020	11	381	1	206	4.9%	5.95 [0.77, 45.74]
Primessnig et al. 2024	13	217	7	217	15.7%	1.86 [0.76, 4.56]
Santos-Martinez et al. 2022	4	125	8	290	11.3%	1.16 [0.36, 3.78]
Voigtländer et al. 2021	11	110	20	288	19.9%	1.44 [0.71, 2.91]
**Total (95% CI)**		**1388**		**2310**	**100.0%**	**1.14 [0.70, 1.85]**
Total events	72		144			

Heterogeneity: $Tau^2 = 0.18$; $Chi^2 = 10.94$, df = 5 (P = 0.05); $I^2 = 54\%$
Test for overall effect: Z = 0.53 (P = 0.60)

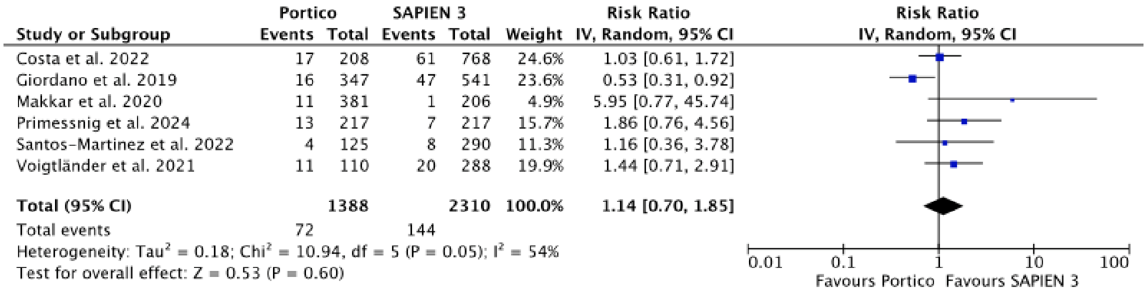

C

Study or Subgroup	Portico Events	Portico Total	SAPIEN 3 Events	SAPIEN 3 Total	Weight	Risk Ratio IV, Random, 95% CI
Costa et al. 2022	2	208	3	768	22.7%	2.46 [0.41, 14.63]
Giordano et al. 2019	0	347	2	541	7.8%	0.31 [0.01, 6.47]
Primessnig et al. 2024	0	217	0	217		Not estimable
Rudolph et al. 2024	4	878	46	13296	69.4%	1.32 [0.48, 3.65]
**Total (95% CI)**		**1650**		**14822**	**100.0%**	**1.36 [0.58, 3.17]**
Total events	6		51			

Heterogeneity: $Tau^2 = 0.00$; $Chi^2 = 1.34$, df = 2 (P = 0.51); $I^2 = 0\%$
Test for overall effect: Z = 0.70 (P = 0.48)

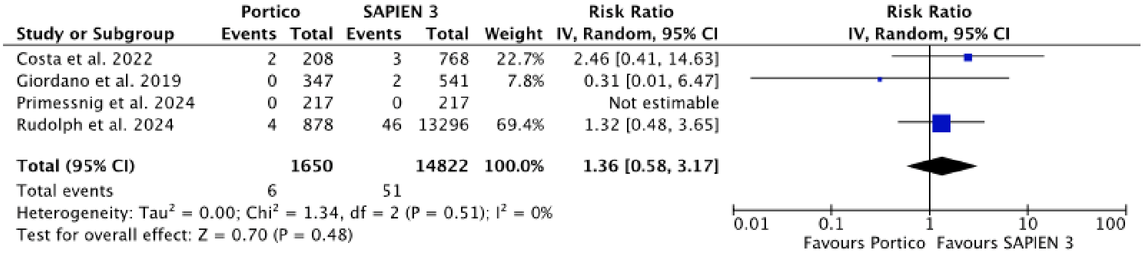

D

Study or Subgroup	Portico Events	Portico Total	SAPIEN 3 Events	SAPIEN 3 Total	Weight	Risk Ratio IV, Random, 95% CI
Brown et al. 2023	1	106	4	176	2.8%	0.42 [0.05, 3.66]
Giordano et al. 2019	8	347	8	541	12.9%	1.56 [0.59, 4.12]
Kim et al. 2017	3	127	12	381	8.2%	0.75 [0.22, 2.62]
Makkar et al. 2020	17	381	0	206	1.7%	18.97 [1.15, 313.78]
Mas-Peiro et al. 2019	6	104	3	73	7.1%	1.40 [0.36, 5.43]
Primessnig et al. 2024	8	217	2	217	5.5%	4.00 [0.86, 18.62]
Rudolph et al. 2024	20	878	313	13296	41.5%	0.97 [0.62, 1.51]
Santos-Martinez et al. 2022	4	125	9	290	9.4%	1.03 [0.32, 3.29]
Voigtländer et al. 2021	5	110	9	288	10.8%	1.45 [0.50, 4.24]
**Total (95% CI)**		**2395**		**15468**	**100.0%**	**1.21 [0.83, 1.76]**
Total events	72		360			

Heterogeneity: $Tau^2 = 0.03$; $Chi^2 = 8.90$, df = 8 (P = 0.35); $I^2 = 10\%$
Test for overall effect: Z = 1.00 (P = 0.32)

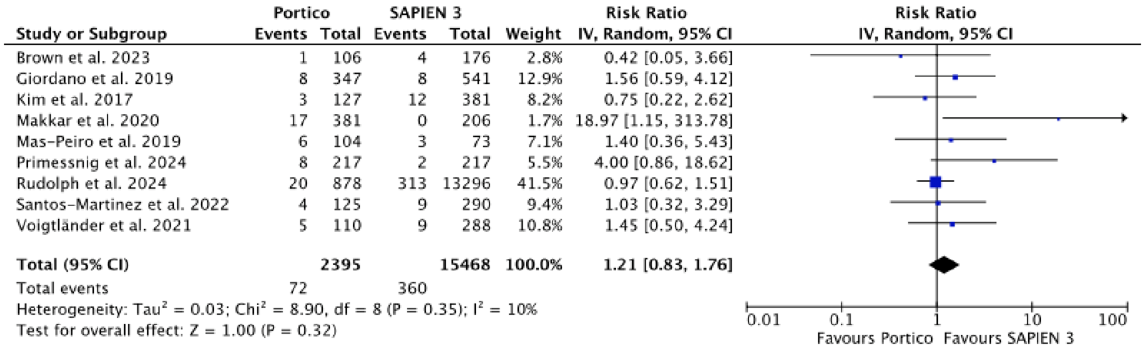

Supplementary Figure S3:

A

Study or Subgroup	Portico Events	Portico Total	SAPIEN 3 Events	SAPIEN 3 Total	Weight	Risk Difference IV, Random, 95% CI
Abdelshafy et al. 2022	11	187	33	397	7.0%	−0.02 [−0.07, 0.02]
Brown et al. 2023	0	106	0	176	14.2%	0.00 [−0.02, 0.02]
Costa et al. 2022	22	208	16	768	7.1%	0.08 [0.04, 0.13]
Giordano et al. 2019	1	347	0	541	15.9%	0.00 [−0.00, 0.01]
Kim et al. 2017	9	127	3	381	6.6%	0.06 [0.02, 0.11]
Leone et al. 2023	27	172	7	286	4.9%	0.13 [0.08, 0.19]
Makkar et al. 2020	21	381	3	206	10.5%	0.04 [0.01, 0.07]
Mas−Peiro et al. 2019	8	104	3	73	3.8%	0.04 [−0.03, 0.10]
Okuno et al. 2020	2	23	9	468	1.5%	0.07 [−0.05, 0.18]
Primessnig et al. 2024	33	217	5	217	5.6%	0.13 [0.08, 0.18]
Rudolph et al. 2024	1	878	17	13296	16.4%	−0.00 [−0.00, 0.00]
Voigtländer et al. 2021	6	110	7	288	6.5%	0.03 [−0.02, 0.08]
**Total (95% CI)**		**2860**		**17097**	**100.0%**	**0.03 [0.02, 0.05]**
Total events	141		103			

Heterogeneity: $Tau^2 = 0.00$; $Chi^2 = 79.41$, df = 11 ($P < 0.00001$); $I^2 = 86\%$
Test for overall effect: Z = 4.05 ($P < 0.0001$)

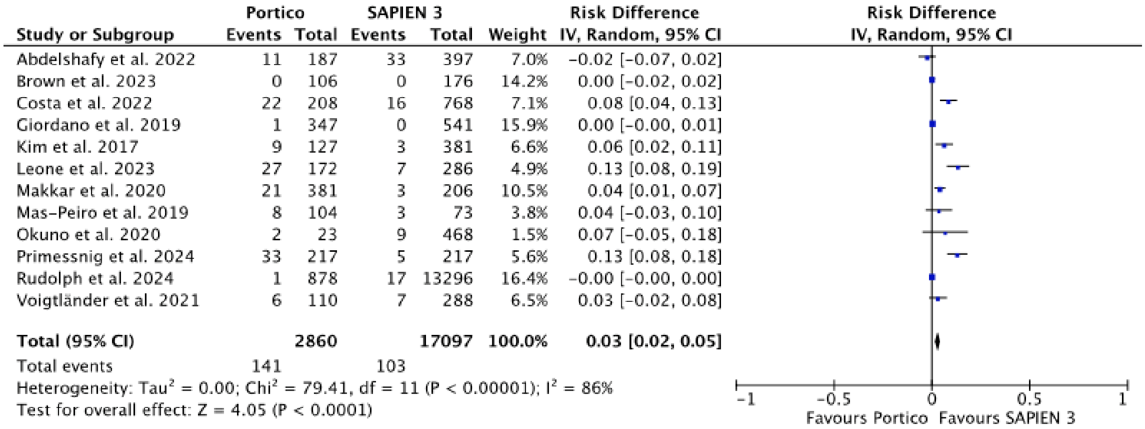

B

Study or Subgroup	Portico Events	Portico Total	SAPIEN 3 Events	SAPIEN 3 Total	Weight	Risk Difference IV, Random, 95% CI
Brown et al. 2023	0	106	0	176	16.0%	0.00 [−0.02, 0.02]
Costa et al. 2022	9	208	25	768	10.9%	0.01 [−0.02, 0.04]
Giordano et al. 2019	17	347	68	541	9.3%	−0.08 [−0.11, −0.04]
Leone et al. 2023	9	172	16	286	7.7%	−0.00 [−0.05, 0.04]
Makkar et al. 2020	36	381	15	206	7.0%	0.02 [−0.02, 0.07]
Mas−Peiro et al. 2019	6	104	4	73	4.0%	0.00 [−0.07, 0.07]
Primessnig et al. 2024	2	217	1	217	15.8%	0.00 [−0.01, 0.02]
Rudolph et al. 2024	78	878	944	13296	14.6%	0.02 [−0.00, 0.04]
Santos−Martinez et al. 2022	3	125	10	290	9.8%	−0.01 [−0.04, 0.02]
Voigtländer et al. 2021	10	110	14	288	5.0%	0.04 [−0.02, 0.10]
**Total (95% CI)**		**2648**		**16141**	**100.0%**	**−0.00 [−0.02, 0.02]**
Total events	170		1097			

Heterogeneity: $Tau^2 = 0.00$; $Chi^2 = 24.50$, df = 9 ($P = 0.004$); $I^2 = 63\%$
Test for overall effect: Z = 0.03 ($P = 0.98$)

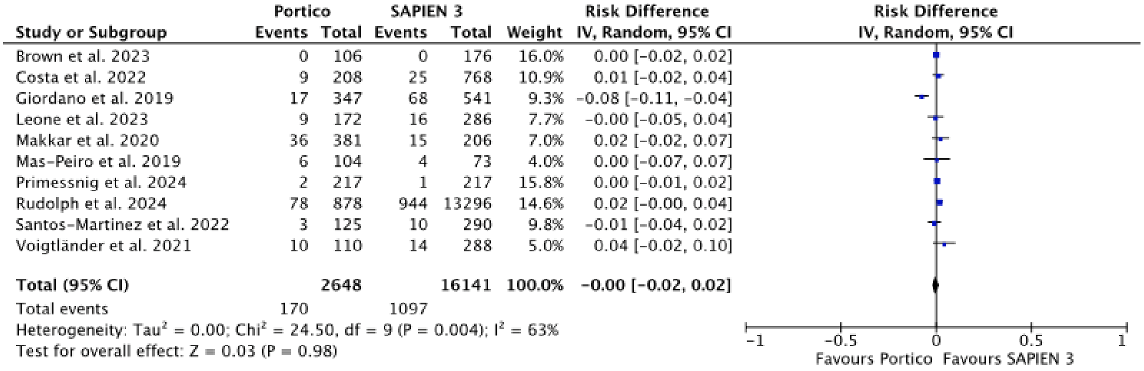

C

Study or Subgroup	Portico Events	Portico Total	SAPIEN 3 Events	SAPIEN 3 Total	Weight	Risk Difference IV, Random, 95% CI
Costa et al. 2022	2	208	3	768	5.9%	0.01 [−0.01, 0.02]
Giordano et al. 2019	0	347	2	541	24.0%	−0.00 [−0.01, 0.00]
Primessnig et al. 2024	0	217	0	217	14.4%	0.00 [−0.01, 0.01]
Rudolph et al. 2024	4	878	46	13296	55.7%	0.00 [−0.00, 0.01]
**Total (95% CI)**		**1650**		**14822**	**100.0%**	**0.00 [−0.00, 0.00]**
Total events	6		51			

Heterogeneity: $Tau^2 = 0.00$; $Chi^2 = 1.95$, df = 3 ($P = 0.58$); $I^2 = 0\%$
Test for overall effect: Z = 0.04 ($P = 0.97$)

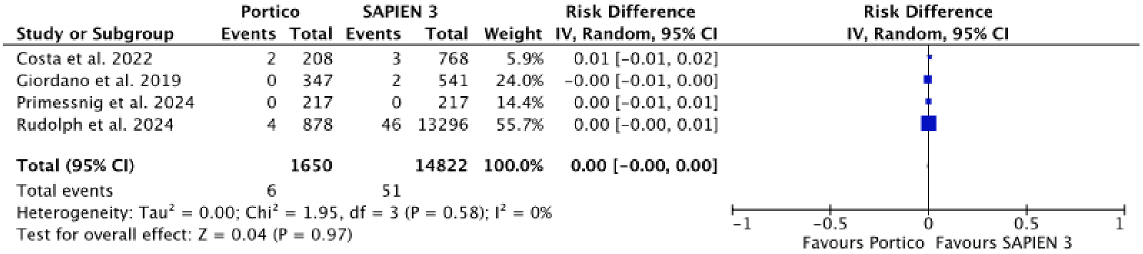

Supplement: Supplementary file 1 [file jcm-15-03573-s001.zip › jcm-4241341-supplementary.pdf]
